# Comparative Analysis of the Subventricular Zone in Rat, Ferret and Macaque: Evidence for an Outer Subventricular Zone in Rodents

**DOI:** 10.1371/journal.pone.0030178

**Published:** 2012-01-17

**Authors:** Verónica Martínez-Cerdeño, Christopher L. Cunningham, Jasmin Camacho, Jared L. Antczak, Anish N. Prakash, Matthew E. Cziep, Anita I. Walker, Stephen C. Noctor

**Affiliations:** 1 Department of Pathology and Laboratory Medicine, School of Medicine, University of California Davis, Sacramento, California, United States of America; 2 Institute for Pediatric Regenerative Medicine, Shriners Hospital for Children of Northern California, Sacramento, California, United States of America; 3 Neuroscience Graduate Program, University of California Davis, Davis, California, United States of America; 4 Department of Biology, Brigham Young University, Rexburg, Idaho, United States of America; 5 Department of Psychiatry, School of Medicine, University of California Davis, Sacramento, California, United States of America; 6 Medical Investigations of Neurodevelopmental Disorders (M.I.N.D.) Institute, School of Medicine, University of California Davis, Sacramento, California, United States of America; Seattle Children's Research Institute, United States of America

## Abstract

The mammalian cerebral cortex arises from precursor cells that reside in a proliferative region surrounding the lateral ventricles of the developing brain. Recent work has shown that precursor cells in the subventricular zone (SVZ) provide a major contribution to prenatal cortical neurogenesis, and that the SVZ is significantly thicker in gyrencephalic mammals such as primates than it is in lissencephalic mammals including rodents. Identifying characteristics that are shared by or that distinguish cortical precursor cells across mammalian species will shed light on factors that regulate cortical neurogenesis and may point toward mechanisms that underlie the evolutionary expansion of the neocortex in gyrencephalic mammals. We immunostained sections of the developing cerebral cortex from lissencephalic rats, and from gyrencephalic ferrets and macaques to compare the distribution of precursor cell types in each species. We also performed time-lapse imaging of precursor cells in the developing rat neocortex. We show that the distribution of Pax6+ and Tbr2+ precursor cells is similar in lissencephalic rat and gyrencephalic ferret, and different in the gyrencephalic cortex of macaque. We show that mitotic Pax6+ translocating radial glial cells (tRG) are present in the cerebral cortex of each species during and after neurogenesis, demonstrating that the function of Pax6+ tRG cells is not restricted to neurogenesis. Furthermore, we show that Olig2 expression distinguishes two distinct subtypes of Pax6+ tRG cells. Finally we present a novel method for discriminating the inner and outer SVZ across mammalian species and show that the key cytoarchitectural features and cell types that define the outer SVZ in developing primates are present in the developing rat neocortex. Our data demonstrate that the developing rat cerebral cortex possesses an outer subventricular zone during late stages of cortical neurogenesis and that the developing rodent cortex shares important features with that of primates.

## Introduction

Neurons of the mammalian cerebral cortex are primarily generated before birth during a period of intense precursor cell proliferation. The number of neurons in the human cortical plate increases by about 5 billion cells between the 13^th^ and 20^th^ weeks of gestation [1], which indicates that on average over 1000 neurons arrive in the CP every second during that seven week period of development. Further, this data suggests that roughly 500 to 1000 precursor cells divide every second to produce cortical neurons during this stage of development. Two principal classes of neural precursor cells have been identified in the developing brain. The primary class of precursor cells resides in the ventricular zone (VZ) adjacent to the lateral ventricle; the secondary class of precursor cells resides in the subventricular zone (SVZ) just superficial to the VZ. In this report we refer to primary precursor cells as radial glial (RG) cells and to secondary precursor cells as intermediate progenitor (IP) cells. RG cells and IP cells can be distinguished based on several characteristics including morphology and the expression of transcription factors. RG cells are bipolar cells that have a ventricular contacting process and a long thin pial process that ascends through the cortical plate to contact the pia via endfeet [2]. RG cells divide at the surface of the ventricle, retain their pial process during division [3]–[5], and express the transcription factor Pax6 [6], [7]. In contrast IP cells are multipolar cells [3]–[5], which in rodent appear to retract all processes during division [4], [5], largely divide away from the surface of the ventricle [5], [8], and express the transcription factor Tbr2 [7].

Rodent studies of cortical development have informed our understanding of mechanisms that regulate prenatal neurogenesis, but recent work has highlighted differences in the development of the rodent and primate cerebral cortices. The SVZ in primates and other gyrencephalic mammals is subdivided into discrete cytoarchitectural regions that are called the inner SVZ (iSVZ) and outer SVZ (oSVZ), while the SVZ in rats and mice is a comparatively thinner structure [9]. Furthermore, the distribution of Pax6+ and Tbr2+ cells is reportedly different in rodents and primates. Pax6+ cells have been described as largely restricted to the VZ in rodents [7], while Pax6+ cells are located in both the VZ and the SVZ in the prenatal cerebral cortex of humans [10], [11], and carnivores such as the ferret [11]. Similarly, Tbr2+ cells are described as largely restricted to the SVZ of rodents [7], but in the human neocortex Tbr2+ cells extend further from the ventricle into the oSVZ [10].

The oSVZ in gyrencephalic mammals is not simply an expanded zone produced by increased numbers of IP cells, but is a distinct zone that includes both IP cells and RG cells that have translocated away from the surface of the ventricle. The presence of translocating RG cells in the developing cortex was initially reported over 30 years ago by Rakic [12]. The morphological transition of RG cells into translocating radial glial cells (tRG cells) has been demonstrated in macaque [12], ferret [13], mouse [14], and human [15]. However, the functional nature of tRG cells had not been determined. Recent studies based on time-lapse imaging of fluorescently labeled cells in live slice cultures have found that tRG cells are present in rodents and play specific functional roles. Noctor and colleagues (2004, 2008), showed time-lapse movies of mitotic translocating RG cells in the embryonic rat neocortex [4], [5], and performed whole-cell patch-clamp recordings of the tRG daughter cells to show that tRG cells produce daughter cells lacking neuronal properties [5]. On the other hand, Miyata and colleagues (2004), showed time-lapse movies of mitotic translocating RG cells in mouse followed by immunohistochemistry and presented evidence that mitotic translocating cells produce daughter neurons based on the expression of the Hu protein [16]. More recent studies have provided evidence that tRG cells express Pax6 and produce daughter neurons in humans [10], and in mice based on expression of the neuronal marker NeuN [17], or lack of mitotic activity [18]. To further explore the characteristics and potential of RG cells, tRG cells, and IP cells in lissencephalic and gyrencephalic mammals and what role they play in the development of gyrencephaly, we compared the cytoarchitecture of the developing cortex in rats, ferrets and macaques, quantified the distribution of Pax6+ and Tbr2+ mitotic cells in each species, and performed time-lapse imaging of green fluorescent protein (GFP) - labeled precursor cells in the embryonic rat cerebral cortex.

We show that most dividing cells in the developing cerebral cortex express Pax6. We show that Pax6+ mitotic cells are located both at the surface of the ventricle and away from the ventricle in each species as described by others, but we note important differences between species. For example, in both lissencephalic rat and gyrencephalic ferret neocortex the majority of mitotic Pax6+ cells are located in the VZ throughout the neurogenic period. In contrast, the distribution of Pax6+ cells in macaque shifts away from the VZ to the oSVZ early in the neurogenic period. We show that Pax6+ translocating radial glial cells are present in the rat cerebral cortex, and that Pax6+ translocating cells are present in the neocortex of each species during and after the period of cortical neurogenesis, demonstrating that Pax6+ translocating cells are not restricted in function solely to neurogenesis. We further characterized Pax6+ cells by costaining for the transcription factors Sox2 and Olig2. Nearly all Pax6+ cells also express Sox2, whereas Olig2 expression distinguishes two subtypes of tRG cells. We show that rats, ferrets and macaques each possess a dense inner band of Tbr2+ cells and a diffuse outer band of Tbr2+ cells. In the macaque these bands correspond precisely with the iSVZ and oSVZ. We also show that the distribution of Tbr2+ cells is more similar in the developing cortex of lissencephalic rats and gyrencephalic ferrets, than it is in the gyrencephalic cortices of macaque and ferret. The macaque exhibits a large shift in the distribution of Tbr2+ cells away from the ventricle, and this occurs much earlier during cortical neurogenesis in macaques than in rats or ferrets. Together these data suggest that the redistribution of Pax6+ and Tbr2+ precursor cells to the oSVZ that occurs in macaque may not be a prerequisite for the development of gyrencephalic cortex since it does not occur in ferret. We show that the inner and outer fiber layers, which can be used to distinguish the boundaries of the iSVZ and oSVZ in macaque visual cortex, are not apparent in somatosensory, motor or frontal cortex. We therefore present a new method for distinguishing the iSVZ and oSVZ in any cortical area that is based on unambiguous histological and immunohistochemical methods. Finally, we propose that the developing rat cerebral cortex has an oSVZ since it possesses the cell types and the cytoarchitectural elements that define the oSVZ in primates.

## Results

The following developmental ages from each species were included in our study. Rats: embryonic day (E)13, E14, E17, E18, E20, E21, E22, postnatal day (P)1, P2, P3, P7 and P10. Mice: E18. Ferrets: E23, E28, E31, E34, E38, P2, P10. Macaques: E50, E65, E80, E100 and E151. These ages included the neurogenic phase beginning with the genesis of layer VI neurons through the genesis of layer II neurons, and the post-neurogenic phase of development in each species [19]–[21]. We analyzed coronal sections of somatosensory cortex except where noted. The number of cells counted for each analysis is included in Table format as noted.

### The distribution of mitotic precursor cells in the developing cortical wall is similar in rats and ferrets, but different in macaques

To investigate the relative contributions of precursor cells in the VZ and SVZ to cortical neurogenesis, we first compared the distribution of mitotic cells in the developing cerebral cortex of rat, ferret and macaque. We identified all mitoses in the dorsal somatosensory cortex in Nissl stained sections from each species and each age encompassing the period of cortical neurogenesis and early stages of gliogenesis. Mitoses were assigned to the VZ, SVZ, iSVZ or oSVZ (when present), or preplate/subplate/cortical plate/marginal zone. We included all mitoses in our analysis, but in this manuscript we focused our attention on precursor cells in the VZ and SVZ. We therefore combined into one bin all preplate, subplate, cortical plate and marginal zone mitoses, even though meaningful differences may exist in the properties of precursor cells within these distinct structures (e.g. [22]). The SVZ first appears during early stages of macaque and ferret cortical development, we label this structure the iSVZ. As development proceeds we distinguished between the iSVZ and oSVZ.

**Figure 1:**
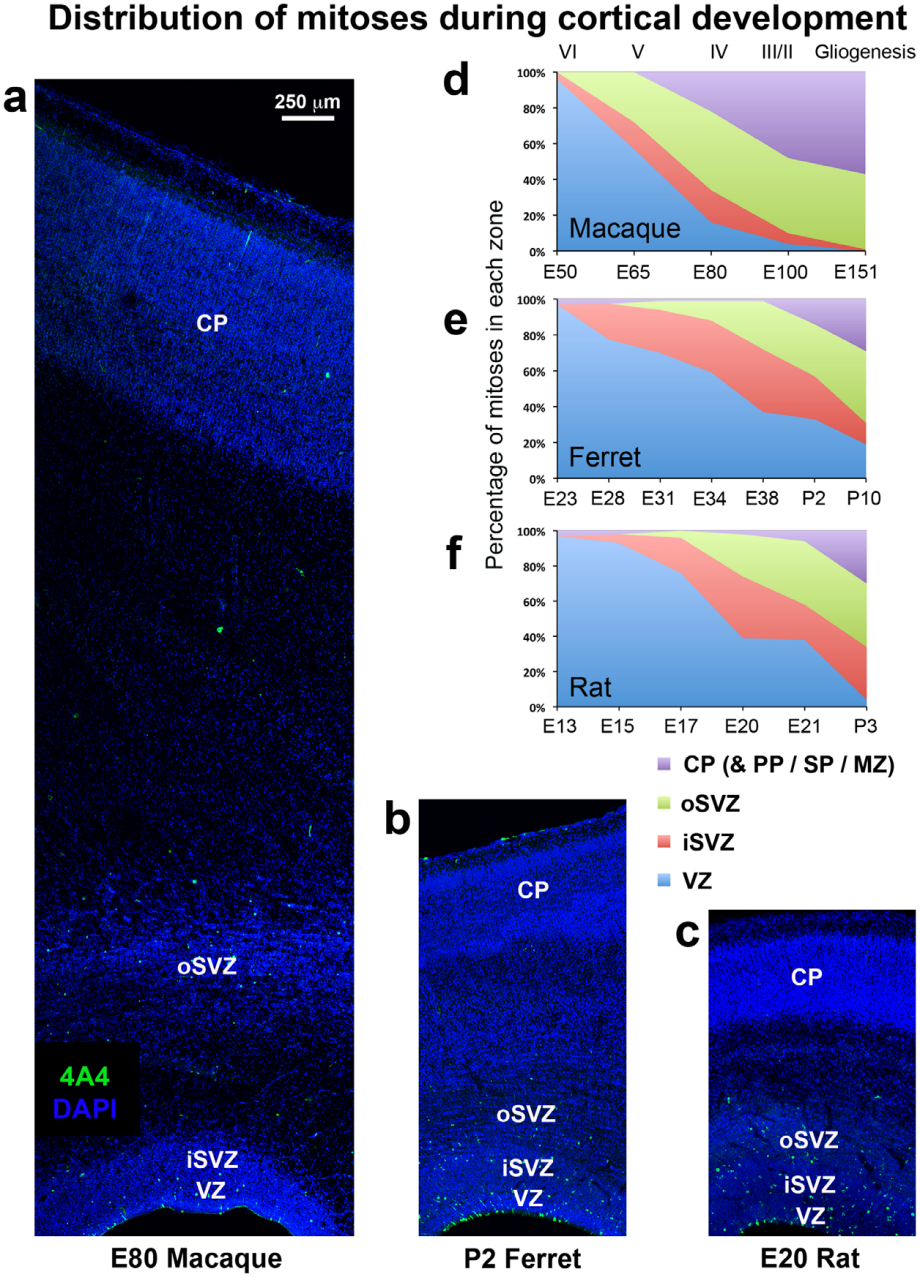
The distribution of mitoses during cortical development is similar in ferrets and rats, but different in macaques. (**a–c**) Coronal sections of somatosensory cortex from E80 macaque, P2 ferret and E20 rat immunostained for phosphorylated vimentin (4A4, green) to label dividing cells and counterstained with DAPI (blue). Images are displayed at the same scale. There are a significant number of abventricular mitoses in each species. (**d–f**) Graphs showing changes in the distribution of mitoses during development of the neocortex. The stage of development is shown at the bottom of the graphs. The approximate cortical layer generated at each stage of development is indicated along the top of the macaque graph, and applies to each graph. (**d**) Early in macaque neurogenesis the majority of mitoses shifted from the ventricular zone (VZ) to abventricular locations. By E80, when layer 4 neurons are being generated [20], less than 20% of mitoses remained in the VZ and 57% were located in the outer subventricular zone (oSVZ). (**e**) The distribution of mitoses in the gyrencephalic ferret did not shift away from the VZ during early stages of neurogenesis as in macaque. At E34 during generation of layer 4 neurons [19], greater than half of all mitoses were still located in the VZ. (**f**) The distribution of mitoses in the lissencephalic rat cortex was similar to that in the gyrencephalic ferret cortex. At E21, which represents the genesis of layer 2 neurons in somatosensory cortex [21], 35% of mitoses were located superficial to the SVZ in a zone that we term the oSVZ. Legend indicates histological zones: VZ: blue; inner SVZ (iSVZ): red; oSVZ: green; cortical plate (CP)/preplate (PP)/subplate (SP)/marginal zone (MZ): purple. Scale bar in (**a**) applies to (**a–c**).

The distribution of mitoses in the developing macaque somatosensory cortex during neurogenesis was dramatically different than that in rat or ferret. We noted a large shift in the distribution of mitoses away from the VZ into the SVZ during early stages of cortical neurogenesis in macaque, but not in rat or ferret (see Table 1 for number of mitoses analyzed). During early stages of cortical development, including stages before the appearance of the SVZ, over 95% of mitoses were located at the surface of the ventricle in each species. In the E50 macaque, at the start of cortical neurogenesis [20], 96% of mitoses were located in the VZ. By E65, during production of layers 5 and 6 neurons in the macaque visual cortex [20], the percentage of mitoses located in the VZ fell to 57%, and by E80, during production of layer 4 neurons [20], the number of mitoses located in the VZ decreased even further to only 16%. In contrast, the number of divisions that were located in the oSVZ of macaque reached 57% at this stage of development. At E100, when production of layer 2 neurons is nearly complete [20], only 4% of mitoses were located in the VZ (Figs. 1a & d).

**Table 1:** Distribution of mitotic cells during neocortical development.

Macaque age	VZ	iSVZ	oSVZ	CP (incl. PP/SP/MZ)
**E50**	95.6% (3603/3759)	4% (141/3759)	0.4% (15/3579)	0% (0/3579)
**E65**	57% (2009/3511)	14.7% (515/3511)	28% (977/3511)	0.3% (10/3511)
**E80**	16% (87/551)	18% (100/551)	44% (242/551)	22% (122/551)
**E100**	4% (22/521)	6% (32/521)	42% (217/521)	48% (250/521)
**E151**	0.2% (2/951)	1% (13/951)	41.8% (397/951)	57% (539/951)

Ferret age	VZ	iSVZ	oSVZ	CP (incl. PP/SP/MZ)
**E23**	96% (377/394)	NA	NA	4% (17/394)
**E28**	78% (538/689)	20% (138/689)	2% (10/689)	0.4% (3/689)
**E31**	70% (447/644)	24% (157/644)	5% (32/644)	1% (8/644)
**E34**	59% (472/802)	28% (228/802)	11% (90/802)	2% (12/802)
**E38**	37% (429/1151)	35% (401/1151)	27% (207/1151)	1% (14/1151)
**P2**	32% (164/509)	24% (121/509)	29% (146/509)	15% (78/509)
**P10**	19% (183/955)	12% (112/955)	40% (386/955)	29% (274/955)

Rat age	VZ	iSVZ	oSVZ	CP (incl. PP/SP/MZ)
**E13**	97% (305/315)	NA	NA	3% (10/315)
**E15**	93% (441/472)	5% (23/472)	NA	2% (8/472)
**E17**	76% (694/912)	20% (185/912)	3.9% (32/912)	0.1% (1/912)
**E20 (4A4)**	39% (145/368)	35% (127/368)	24% (88/368)	2% (8/368)
**P3 (4A4)**	4% (23/520)	30% (155/520)	36% (186/520)	30% (156/520)

Numbers represent the proportion of mitotic cells in each compartment of the developing somatosensory cortex in each species. Cells were counted in a minimum of seven coronal sections from each age in each species. Mitoses were identified by 4A4 immunoreactivity, or condensed chromatin using DAPI or Nissl staining. Numbers in parentheses indicate the total number of mitotic cells counted in each compartment for each age in each species.

In the developing ferret somatosensory cortex the majority of divisions remained in the VZ throughout most of the neurogenic period. At E23, the beginning of cortical neurogenesis [19], the percentage of cortical divisions located in the VZ was 96%. At E28 the percentage of mitoses within the VZ was 78%, and by E34, during the production of layer 4 neurons in somatosensory cortex [19], the majority of divisions were still located in the VZ (59%). By P2, when production of layer 2 neurons is nearly complete in ferret somatosensory cortex [19], the percentage of mitoses in the VZ decreased to 32%. At P10 when neurogenesis was complete in ferret somatosensory cortex [19], the percentage of VZ mitoses had decreased to 9% (Figs. 1b & e).

In the developing rat somatosensory cortex the distribution of mitoses was very similar to that of ferret. At E13 in the rat 97% of divisions were in the VZ. At E17, which is the peak of neurogenesis for layer 4 neurons in rat somatosensory cortex [21], over 75% of all mitoses were still located in the VZ. At E21, when the vast majority of layer 2 neurons in rat somatosensory cortex have been generated [21], the percentage of mitoses located in the VZ had decreased to 38% and the majority of divisions were located in the SVZ. By P3, when cortical neurogenesis is complete in rat somatosensory cortex [21], the percentage of VZ mitoses had dropped to 4% (Figs. 1c & f).

Rat and ferret also shared a similar pattern in the number of mitoses away from the ventricle. The percentage of mitoses in the SVZ of the rat and in the iSVZ of the ferret began increasing at the onset of neurogenesis and reached a peak of ∼30% during neurogenesis of layer 2 neurons. The proportion of divisions in the ferret oSVZ reached 29% by P2.

During later stages of rat cortical development we noted that a substantial proportion of divisions occurred superficial to the SVZ, as defined by the Boulder Committee [23]. These divisions were located between the SVZ and the subplate. We have previously shown that some mitotic intermediate progenitor cells produce neurons in this zone [5]. We found that the proportion of mitoses in this zone of developing rat cortex reached 24% by E20, during neurogenesis of layer two neurons. In addition, the number of divisions occurring in the subplate/cortical plate/marginal zone began increasing during genesis of layer 2 neurons and reached 30% in rat and ferret in the post neurogenic developing cortex.

These data demonstrate that the distribution of mitoses in the cortical wall is more similar in the lissencephalic rat and the gyrencephalic ferret, than the distribution of mitoses in the gyrencephalic cortices of ferret compared to that of the macaque. These data suggest that the large increase in the proportion of mitoses located away from the ventricle that occurs during early stages of macaque neurogenesis may not be a prerequisite for the development of gyrencephalic cortex since it does not occur in ferret.

### The distribution of Tbr2-expressing cells is similar in rats and ferrets, but different in macaques

**Figure 2:**
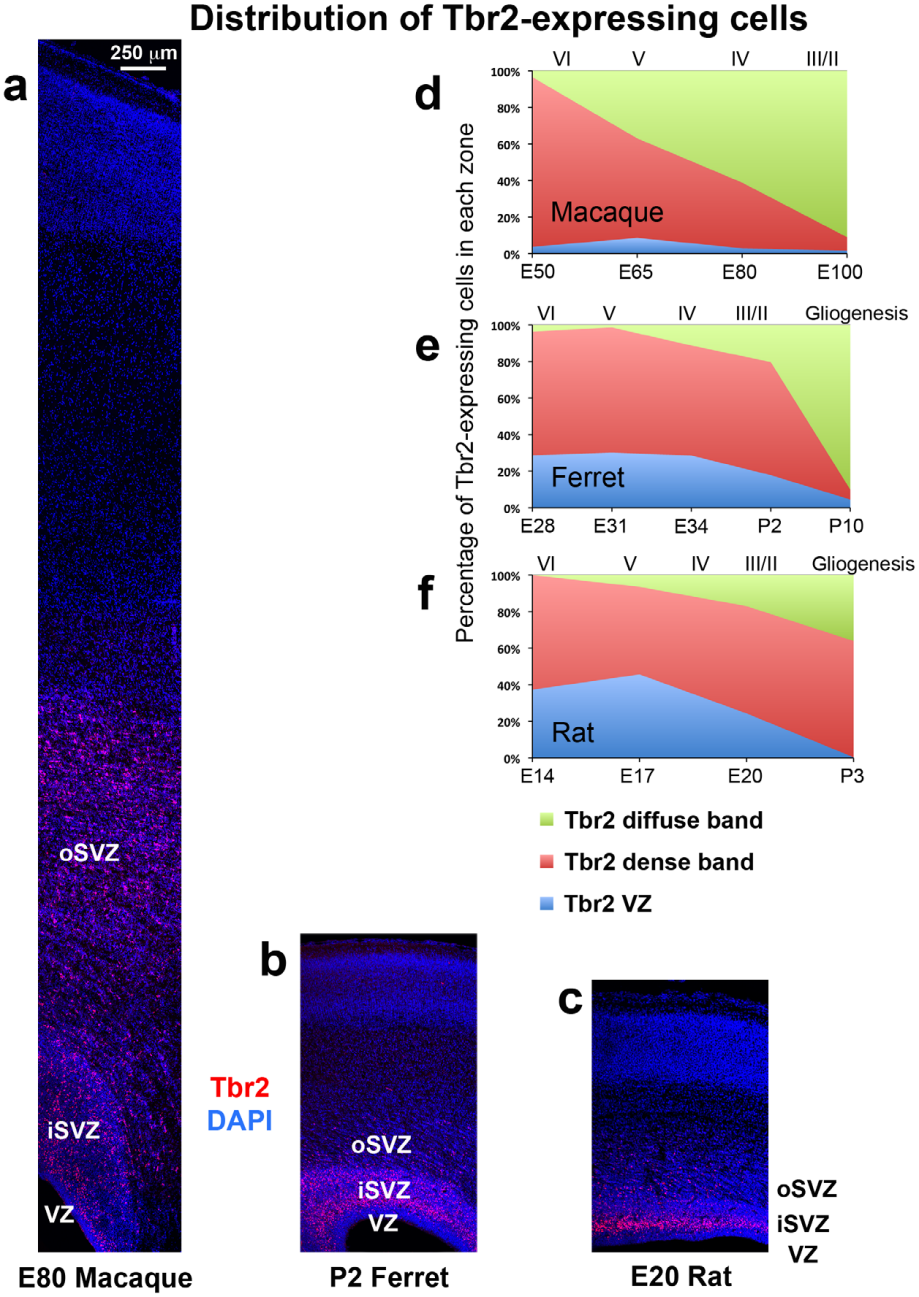
The distribution of Tbr2+ cells during cortical development is similar in ferrets and rats, but different in macaques. (**a–c**) Coronal sections of somatosensory cortex from E80 macaque, P2 ferret and E20 rat immunostained for Tbr2 (red) and counterstained with DAPI (blue). Images are displayed at the same scale. In each species a dense inner band of Tbr2+ cells was located within the cell dense zone surrounding the lateral ventricle visualized with DAPI. The diffuse outer band of Tbr2 expression was located within a striated zone marked by streams of cells organized in tangential clusters. (**d–f**) Graphs showing changes in the distribution of Tbr2+ cells during development of the cerebral cortex. The stage of development is shown at the bottom of the graphs, and the approximate cortical layer generated at each stage of development is indicated along the top of each graph. (**d**) In macaque the distribution of Tbr2+ cells progressively shifted to the outer subventricular zone (oSVZ) during development. By E80, during genesis of layer 4 neurons [20], 59% of Tbr2+ cells were located in the oSVZ. (**e**) The distribution of Tbr2+ cells in the gyrencephalic ferret shifted to the oSVZ more slowly in comparison to macaque. At P2 during genesis of layer 2 neurons [19], 62% of Tbr2+ cells remained in the inner SVZ (iSVZ). (**f**) The distribution of Tbr2+ cells in the lissencephalic rat was similar to that of the gyrencephalic ferret. At E20 during genesis of upper layer neurons [21], the majority of Tbr2+ cells (59%) were located in the iSVZ, and 17% were located in the oSVZ. Legend indicates histological zones: VZ: blue; iSVZ: red; oSVZ: green. Scale bar in (**a**) applies to (**a–c**).

The transcription factors Pax6 and Tbr2 are commonly used to identify and to distinguish VZ and SVZ precursor cells [6], [7]. To characterize SVZ precursor cells in lissencephalic and gyrencephalic mammals we examined the distribution of Tbr2+ cells in each species. Previous reports have examined Tbr2 expression in lissencephalic rodents [7], and gyrencephalic mammals including ferrets [11], and humans [10]. We first qualitatively compared the expression patterns of Tbr2 in each species. The onset of Tbr2 expression occurred prior to the appearance of the SVZ in mammalian cortex. The first Tbr2 cells were dispersed throughout the VZ, but as development proceeded Tbr2+ cells were concentrated in a dense band superficial to the VZ, thus contributing to the formation of the SVZ [5]. The dense band of Tbr2 expression remained in the dorsal cortex of rat, ferret and macaque throughout most of cortical development, and soon after a diffuse outer band of Tbr2+ cells appeared superficial to the dense inner band. The dense inner band of Tbr2 expression was located within a cell dense proliferative region that surrounds the lateral ventricle during cortical development, and can be visualized in Nissl or DAPI stained tissue (Figs. 1 and 2). The diffuse outer band of Tbr2 expression was located in a cellular region characterized by lower cell density and by tangential or obliquely oriented streams of cells that give a striated or stippled appearance to this zone of the developing cortex. To understand the dynamics of SVZ precursor cell distribution during cortical development we quantified the distribution of Tbr2+ cells across neurogenic stages in the three species. We identified all Tbr2+ cells in 200 µm wide radial bins of somatosensory cortex that spanned from the ventricle to the pia in coronal sections from each species and quantified the proportion of immunopositive cells located in each lamina of the developing cortex. We found more similarities in the distribution of Tbr2+ cells in the cortex of the lissencephalic rat and gyrencephalic ferret, than we did in the distribution of Tbr2+ cells in the developing gyrencephalic cortices of ferret and macaque (see Table 2 for number of Tbr2+ cells analyzed).

**Table 2:** Distribution of Tbr2+ cells during neocortical development.

Macaque age	Total Tbr2+ cells	Radial units	VZ	Tbr2+ dense inner band	Tbr2+ diffuse outer band
**E50**	267	3	4% (10/267)	93% (248/267)	3% (9/267)
**E65**	720	3	9% (66/720)	50% (361/720)	41% (293/720)
**E80**	1213	3	2% (29/1213)	36% (433/1213)	62% (751/1213)
**E100**	520	3	2% (10/520)	8% (42/520)	90% (468/520)

Ferret age	Total Tbr2+ cells	Radial units	VZ	Tbr2+ dense inner band	Tbr2+ diffuse outer band
**E28**	290	3	29% (84/290)	68% (196/290)	3% (10/290)
**E31**	464	3	30% (140/464)	68% (317/464)	2% (7/464)
**E34**	456	3	28% (128/456)	60% (275/456)	12% (53/456)
**P2**	719	3	18% (129/719)	62% (444/719)	20% (146/719)
**P10**	81	3	5% (4/81)	6% (5/81)	89% (72/81)

Rat age	Total Tbr2+ cells	Radial units	VZ	Tbr2+ dense inner band	Tbr2+ diffuse outer band
**E14**	245	3	40% (97/245)	60% (148/245)	NA
**E17**	3439	16	46% (1464/3214)	48% (1545/3214)	6% (205/3214)
**E20**	516	3	24% (123/516)	59% (304/516)	17% (89/516)
**P3**	243	3	0.4% (1/243)	63.6% (154/243)	36% (88/243)

Tbr2+ cells were counted in a minimum of three 200 µm wide radial units from each age in each species. Radial units stretched from the ventricle to the pial surface in dorsal somatosensory cortex. Numbers represent the average percentage of Tbr2+ cells in each compartment averaged across *n* radial units. Numbers in parentheses indicate the total number of Tbr2+ cells counted in each compartment at each age.

The age of onset for Tbr2 expression in macaque has not yet been determined. At E50 in the macaque 96% of Tbr2+ cells were located in the dense band of Tbr2 expression. Histologically, the oSVZ was not discernible in the cortical wall at this point of macaque development. By E65, during generation of layers 5 and 6 [20], both the dense inner band and diffuse outer band of Tbr2 expression were apparent. We found that 54% of Tbr2+ cells were located in the dense inner band, and that 37% of Tbr2+ cells were located in the diffuse outer band. The proportion of Tbr2+ cells located in the diffuse outer band of macaque cortex increased as cortical development proceeded. At E80, the majority of Tbr2+ cells were located in the diffuse outer band (59%), and by E100, which corresponds to the end of neurogenesis for layer 2 cortical neurons [20], 91% of Tbr2+ cells were located in the diffuse outer band and only 7% of Tbr2+ cells remained in the dense inner band (Figs. 2a & d).

**Figure 3:**
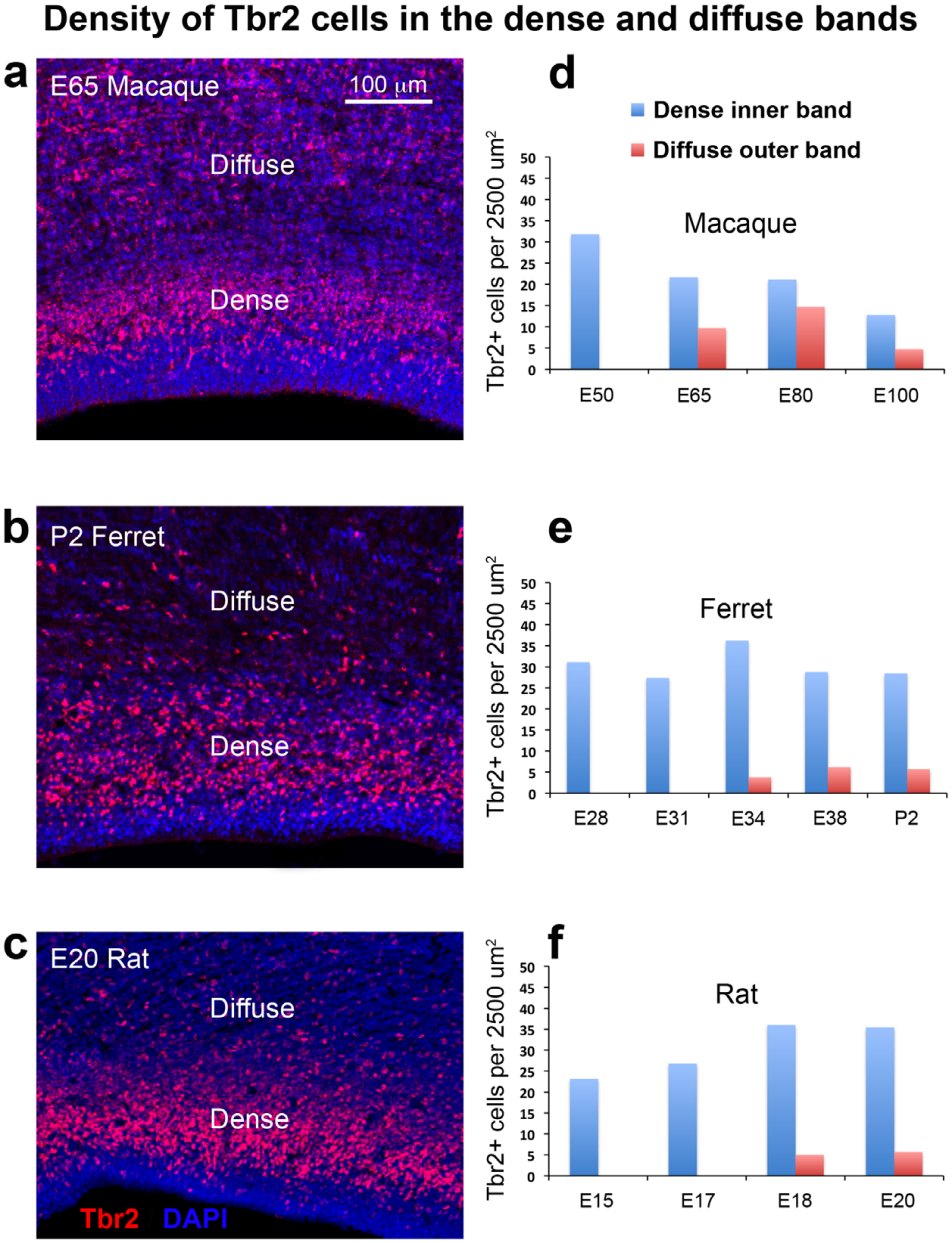
Density of Tbr2+ cells in the dense inner band and diffuse outer band of macaque, ferret and rat. (**a–c**) Images taken from coronal sections of E65 macaque, P2 ferret and E20 rat immunostained for Tbr2 (red) and counterstained with DAPI (blue). The dense inner band and diffuse outer band of Tbr2+ cells are indicated. (**d–f**) Histograms displaying the number of Tbr2+ cells per 2500 µm^2^ in the dense inner band (blue) and diffuse outer band (red) for each species. The diffuse outer band of Tbr2+ cells appears at an early age in macaque in comparison to ferret and rat. In macaque the diffuse outer band was present at E65 during generation of deep layer neurons while the diffuse outer band first appeared at later stages of development in ferret and rat. Nonetheless, the relative density of Tbr2+ cells in the dense and diffuse bands was similar in each species. Scale bar in (**a**) applies to (**a–c**).

We noted a significant difference in the density of Tbr2+ cells in the dense inner band versus the diffuse outer band of the SVZ. At E65 Tbr2+ cells were concentrated at 25 cells per 2500 µm^2^ in the dense inner band, and approximately 10 cells per 2500 µm^2^ in the diffuse outer band of the SVZ (Fig. 3a). The density of Tbr2+ cells in the dense inner band and diffuse outer band were fairly constant throughout much of development. But by E100, at the end of cortical neurogenesis, the dense inner band was reduced in thickness and the density of Tbr2+ cells dropped in both the dense inner band and diffuse outer band (Fig. 3a, Table 3).

**Table 3:** Density of Tbr2+ cells per 2500 mm^2^ in dense inner band and diffuse outer band.

Macaque age	Total Tbr2+ cells counted	Density of dense inner band	Density of diffuse outer band
**E50**	286	32	NA
**E65**	784	25	12
**E80**	322	21	15
**E100**	157	13	5

Ferret age	Total Tbr2+ cells counted	Density of dense inner band	Density of diffuse outer band
**E28**	280	31	NA
**E31**	246	27	NA
**E34**	423	36	4
**E38**	390	29	6
**P2**	535	28	6

Rat age	Total Tbr2+ cells counted	Density of dense inner band	Density of diffuse outer band
**E14**	208	23	NA
**E17**	307	27	NA
**E18**	138	36	5
**E20**	803	35	6

Density measurements are average number of Tbr2+ cells in the dense inner band and diffuse outer band counted in at least three 2500 µm^2^ regions of interest at each age in each species. Bands were identified by the pattern of Tbr2 immunoreactivity.

**Figure 4:**
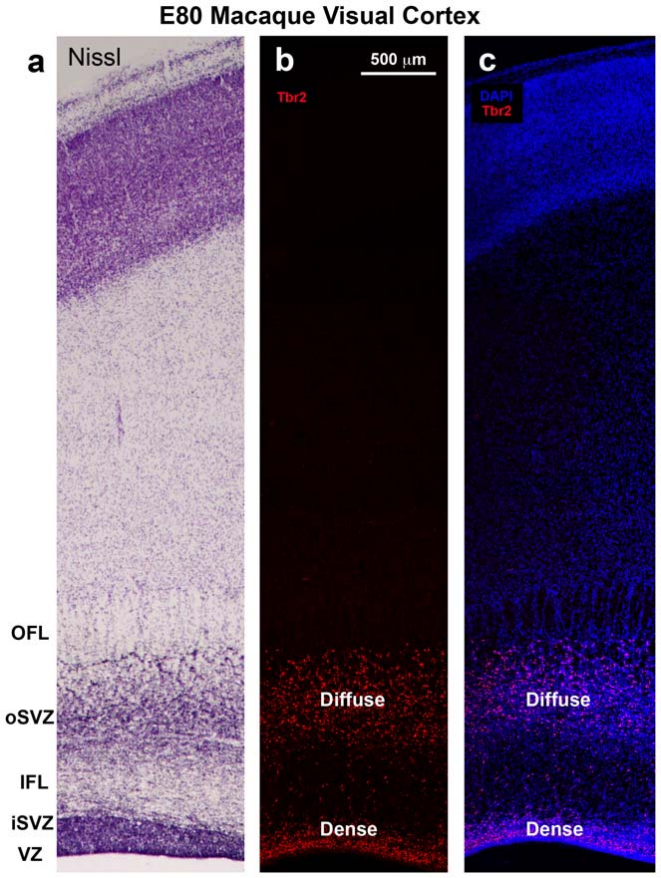
The dense inner band of Tbr2+ cells corresponds to the inner subventricular zone (iSVZ) and the diffuse outer band of Tbr2+ cells corresponds to the outer SVZ (oSVZ) in macaque visual cortex. (**a**) Nissl stained E80 macaque visual cortex. (**b**) An adjacent section immunostained for Tbr2 (red). The dense inner band and diffuse outer band are indicated. (**c**) Image of the same adjacent section showing Tbr2 (red) and DAPI stain (blue). The different compartments, such as the outer fiber layer (OFL) can be visualized with DAPI stain. VZ, ventricular zone; IFL, inner fiber layer. Scale bar in (**b**) applies to (**a–c**).

We next asked if the dense inner band and diffuse outer band of Tbr2+ cells correspond to the inner and outer SVZ. We compared the pattern of Tbr2 expression with cortical cytoarchitecture on adjacent Tbr2/DAPI and Nissl stained sections of the macaque neocortex in the occipital, parietal and frontal lobes. The inner and outer SVZ were identified following the conventions of Smart and colleagues, namely radial organization of cells in the oSVZ and more randomly organized cells in the iSVZ [9] (Fig. 4a). Side by side comparison of Nissl and Tbr2 staining in visual cortex confirmed that the dense inner band of Tbr2 expression corresponded to the iSVZ and that the diffuse outer band of Tbr2 expression corresponded to the oSVZ (Figs. 4a & b).

**Figure 5:**
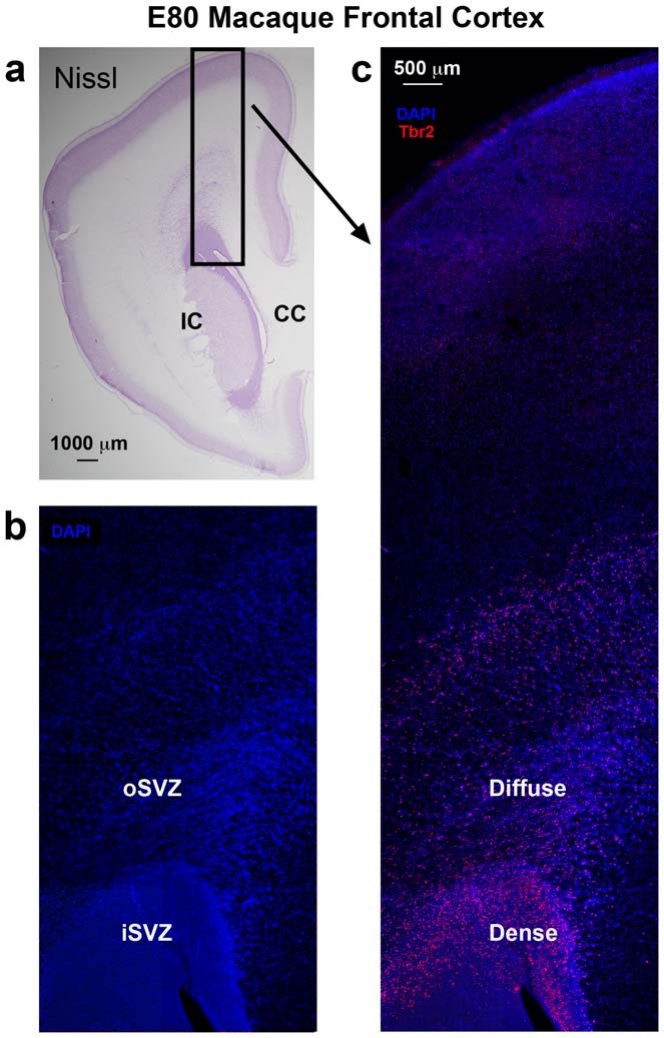
Inner and outer fiber layers are not apparent in macaque cortical areas rostral to the occipital lobe. (**a**) Nissl stained coronal section of E80 macaque frontal lobe at the level of the genu of the corpus callosum (CC). (**b–c**) An adjacent section taken from the E80 macaque frontal lobe stained with DAPI (blue) and Tbr2 (red). (**b**) DAPI staining highlights the different cell densities within the inner subventricular zone (iSVZ) and outer SVZ (oSVZ). The cell dense region surrounding the lateral ventricle includes the very thin ventricular zone and the iSVZ. Tangential streams of cells can be visualized within the oSVZ in DAPI staining. Inner and outer fiber layers are not apparent. (**c**) The same section showing Tbr2 (red) and DAPI. As in visual cortex, the dense inner band of Tbr2 cells corresponds to the iSVZ and the diffuse outer band of Tbr2+ cells corresponds to the oSVZ. Scale bar in (**c**) applies to (**b**). IC, internal capsule.

We also noted that it was possible to distinguish the inner and outer SVZ, and the inner and outer fiber layers by examining the pattern of DAPI staining (Fig. 4c). The outer fiber layer (OFL) serves as the upper boundary of the SVZ in macaque visual cortex and can be visualized in both Nissl and DAPI stained tissue by the striking presence of radial streams of cells (Fig. 4). Tbr2 cells do not penetrate into the macaque OFL. The inner fiber layer of macaque visual cortex lies between the iSVZ and oSVZ, and can be visualized in DAPI or Nissl stained tissue by a marked decrease in cell density. In Tbr2 stained tissue the inner fiber layer can be visualized as a gap between the dense inner band and diffuse outer band of Tbr2 expression (Fig. 4b). However, in cortical areas rostral to visual cortex, the inner fiber layer was not apparent, there was no separation between the iSVZ and oSVZ, and no separation between the dense inner band and diffuse outer band of Tbr2+ cells (Fig. 5). In other cortical areas it was possible to distinguish the iSVZ from the oSVZ by examining the pattern of Nissl or DAPI staining. The boundary between the iSVZ and oSVZ can be discriminated as a very sharp border created by differences in cell density (Figs. 5a & b). The VZ, iSVZ and the Tbr2 dense inner band were located in the cell dense region that surrounds the lateral ventricle. The oSVZ was located in a region that has a lower cell density compared to the iSVZ, but a higher cell density than the overlying intermediate zone (Figs. 5a & c). We noted that the diffuse outer band of Tbr2 staining/oSVZ extended dorsally two to three times farther from the lateral ventricle in somatosensory cortex than it does in visual cortex (Figs. 4 and 5).

In ferret we also noted the presence of a dense inner band of Tbr2 expression during early stages of cortical development, and the addition of a diffuse outer band of Tbr2 expression at later stages. The dense inner band and diffuse outer band of Tbr2 expression in ferret also corresponded to the iSVZ and oSVZ. The boundary between the iSVZ and oSVZ in ferret could also be discriminated in DAPI staining as a sharp border created by high cell density in the iSVZ and lower cell density in the oSVZ. However, the developmental pattern of Tbr2 expression in ferret differed from that in macaque. Whereas the majority of Tbr2+ cells shifted to the oSVZ early in macaque development, in ferret the majority of Tbr2+ cells (>60%) remained in the iSVZ throughout the entire period of cortical neurogenesis, and a much smaller proportion of Tbr2+ cells were located in the oSVZ. In ferret the proportion of Tbr2+ cells located in the oSVZ was only 2% at E31, when layer 5 neurons are generated [19], 11% at E34, when layer 4 neurons are generated [19], and 20% at P2 (Figs. 2b & e), which corresponds to the end of cortical neurogenesis in ferret somatosensory cortex [19]. We quantified the density of Tbr2+ cells in the ferret iSVZ and oSVZ and found a similar trend to the density of Tbr2+ cells in the macaque: much greater density in the iSVZ compared to the oSVZ (Fig. 3b & e).

Inner and outer subdivisions of the SVZ have not been described in rodents as they have for macaque and ferret. Nonetheless, we also noted two distinct patterns of Tbr2 distribution in the rat: a dense inner band of Tbr2-expressing cells during early stages of cortical development, and the addition of a diffuse outer band of Tbr2-expressing cells at later stages. As in macaque and ferret the border between the inner dense band and outer diffuse band of Tbr2 staining could be discriminated in DAPI stained tissue as a sharp border created by differences in cell density, but only after E17. At the earliest stages of rat cortical development the diffuse outer band was either not present or contained a very small proportion of the total number of Tbr2+ cells in radial bins of the dorsal cortex. At E17 only 6% of Tbr2+ cells were located in the diffuse outer band of the SVZ, but by E20, when the majority of layer 2 neurons have been generated [21], the percentage of Tbr2+ cells in the diffuse outer band of the SVZ rose to 17% (Figs. 2c & f). We quantified the density of Tbr2+ cells in the dense inner band and the diffuse outer band of the rat SVZ and found that it was similar to the density of Tbr2+ cells in ferret and macaque. The density of Tbr2+ cells was 25 to 35 cells per 2500 µm^2^ in the dense inner band of the SVZ and five to 10 cells per 2500 µm^2^ in the diffuse outer band of the SVZ (Fig. 3c & f).

**Figure 6:**
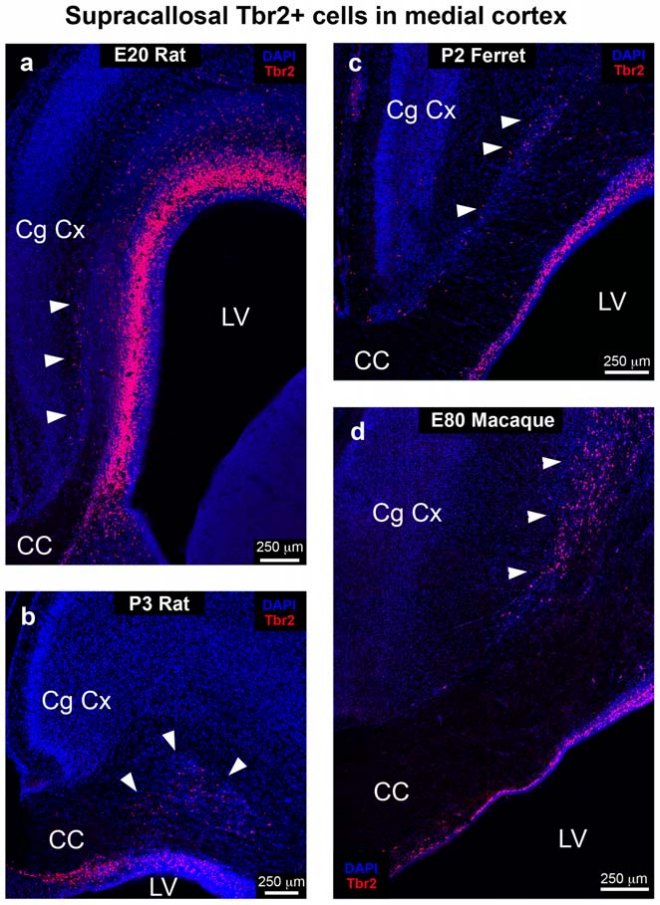
Tbr2+ cells are located superficial to the developing white matter near the corpus callosum (CC) in the prenatal medial cortex of macaque, ferret and rat. (**a–d**) Coronal sections from rat, ferret, and macaque immunostained for Tbr2 (red) and counterstained with DAPI (blue). (**a**) Image from E20 rat showing clusters of Tbr2+ cells superficial to developing callosal fibers along the medial wall of the cortex (arrowheads). (**b**) Image from P3 rat showing an isolated pocket of Tbr2+ cells (arrowheads) located deep within the developing cingulate cortex (Cg Cx) superficial to the developing white matter. (**c**) Tbr2+ cells located superficial to the developing callosal fibers along the medial wall of the P2 ferret cortex (arrowheads). (**d**) Tbr2+ cells positioned above callosal fibers (arrowheads) in the cingulate cortex of E80 macaque. The supracallosal Tbr2+ cells appear to be oSVZ cells that were separated from underlying precursor cells by growing callosal axons. LV, lateral ventricle.

In the rat cortex during late stages of development we observed a cluster of Tbr2+ cells in the cingulate cortex that were located within and superficial to callosal fibers (Fig. 6). These Tbr2+ cells were apparent at P3 (Fig. 6b) but no longer present at P10. We noted Tbr2+ cells located in the same position in ferret and macaque cingulate cortex (Figs. 6c & d). The supracallosal Tbr2+ cells in ferret and macaque appeared to be oSVZ cells that were separated from the underlying precursor cells by growing callosal axons. The position of Tbr2+ cells in the rat cingulate cortex appeared remarkably similar to that in ferret and macaque.

**Figure 7:**
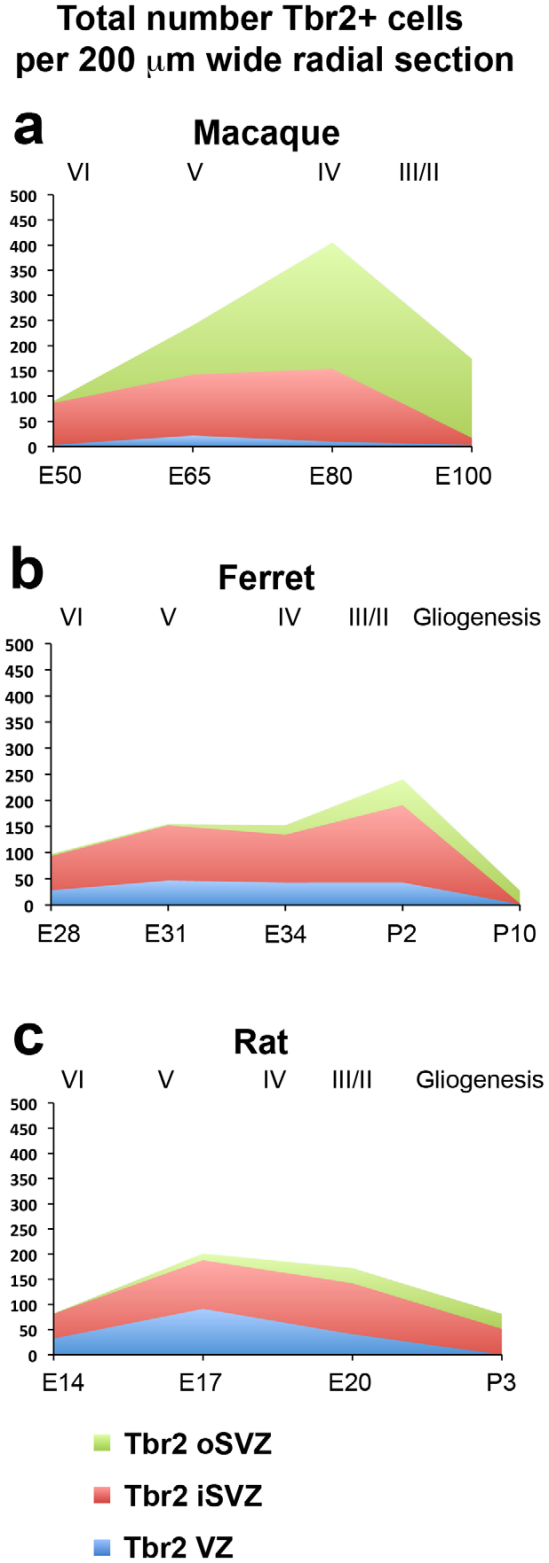
Graphs showing the total number of Tbr2+ cells in the ventricular zone (VZ), inner subventricular zone (iSVZ) and outer SVZ (oSVZ) within a 200 µm wide radial unit of macaque, ferret and rat somatosensory cortex. (**a–c**) There was a similar number of Tbr2+ cells in the iSVZ of each species, but macaque had a much larger number of Tbr2+ cells in the oSVZ. The stage of development is shown at the bottom of each graph, and the approximate cortical layer generated during each stage of development is indicated along the top of each graph. Legend indicates histological zones: VZ: blue; iSVZ: red; oSVZ: green.

**Figure 8:**
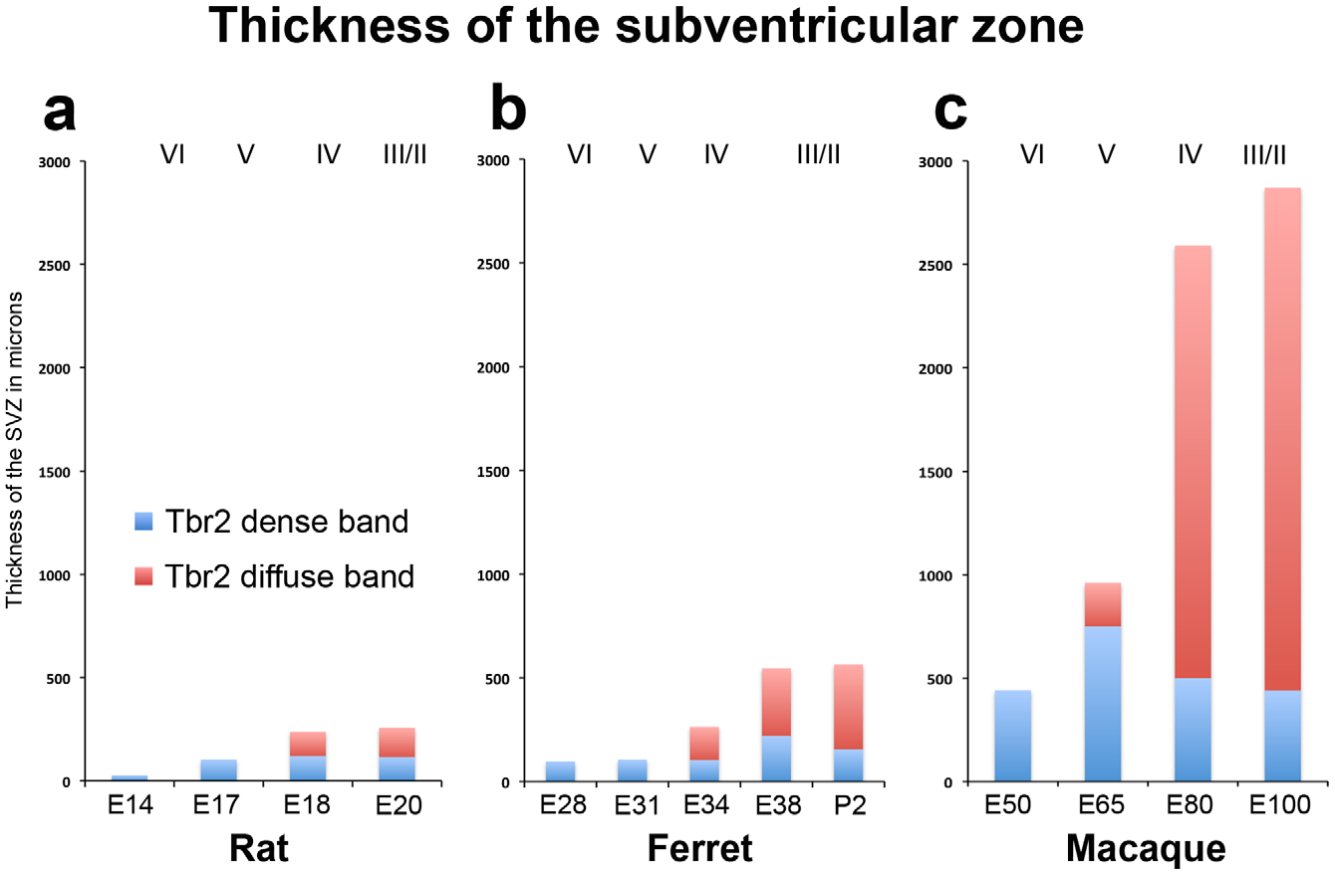
Histograms comparing the thickness of the subventricular zone in the developing cortex of rat, ferret, and macaque. Measurements of the thickness of the dense inner band (blue) and diffuse outer band (red) of Tbr2+ cells were made in radial bins stretching from the ventricle to the pial surface in the dorsal somatosensory cortex of rat (**a**), ferret (**b**), and macaque (**c**). In each species the dense inner band appeared first in development followed by the diffuse outer band. In macaque the dense inner band and diffuse outer band were both significantly thicker than in ferret or rat. The stage of development is shown at the bottom of the graphs, and the approximate cortical layer generated at each stage of development is indicated along the top of each graph.

We compared the total number of Tbr2+ cells in each lamina of the developing cortex of each species within 200 µm wide radial sections (see Table 4 for numbers). This analysis showed that there was a similar number of cells in the iSVZ of each species per 200 µm radial bin (100 to 150 cells), but that macaque possessed a far greater number of Tbr2+ cells in the oSVZ than either rats or ferrets (Fig. 7). Furthermore, the thickness of the Tbr2 diffuse band/oSVZ in developing somatosensory cortex in macaque was much greater than in either rat or ferret (Fig. 8, Table 5).

**Table 4:** Average number of Tbr2+ cells in the VZ, iSVZ and oSVZ/200 µm radial unit during neocortical development.

Macaque age	Average Tbr2+ cells/200 µm bin	Radial units	VZ	iSVZ	oSVZ
**E50**	89	3	3	83	3
**E65**	240	3	22	120	98
**E80**	404	3	10	144	250
**E100**	173	2	3	14	156

Ferret age	Average Tbr2+ cells/200 µm bin	Radial units	VZ	iSVZ	oSVZ
**E28**	97	3	28	66	3
**E31**	155	3	47	106	2
**E34**	152	3	43	92	17
**P2**	240	2	43	148	49
**P10**	27	3	1	2	24

Rat age	Average Tbr2+ cells/200 µm bin	Radial units	VZ	iSVZ	oSVZ
**E14**	82	3	32	50	0
**E17**	202	12	92	97	13
**E20**	172	3	41	101	30
**P3**	81	3	0	51	30

Numbers represent the average number of Tbr2+ cells in each compartment of developing somatosensory cortex per radial unit averaged across *n* radial units. Cells were counted in a minimum of two 200 µm wide radial units from each age in each species. Cells were identified through Tbr2 immunoreactivity.

**Table 5:** Radial thicknesses of the SVZ, the Tbr2+ dense inner band and the Tbr2+ diffuse outer band.

Macaque age	Total SVZ	Dense inner band	Diffuse outer band
**E50**	440 µm	440 µm	NA
**E65**	960 µm	750 µm	210 µm
**E80**	2590 µm	500 µm	2090 µm
**E100**	2870 µm	440 µm	2430 µm

Ferret age	Total SVZ	Dense inner band	Diffuse outer band
**E28**	97 µm	97 µm	NA
**E31**	105 µm	105 µm	NA
**E34**	263 µm	103 µm	160 µm
**E38**	545 µm	220 µm	325 µm
**P2**	563 µm	156 µm	407 µm

Rat age	Total SVZ	Dense inner band	Diffuse outer band
**E14**	27 µm	27 µm	NA
**E17**	103 µm	103 µm	NA
**E18**	237 µm	120 µm	117 µm
**E20**	257 µm	117 µm	140 µm

Radial thickness from ferret and rat are averages obtained from a minimum of two radial units per age. Radial thicknesses from macaque are from one radial unit per age. The dense inner band and diffuse outer band were identified by the pattern of Tbr2 immunoreactivity.

The proportion of Tbr2+ cells located in the diffuse outer band of the SVZ, or oSVZ, at the end of cortical neurogenesis was similar in ferret (20%) and rat (17%), but dramatically higher in macaque (>60%). These results provide two notable findings. First, the shift in the distribution of Tbr2+ cells to the outer SVZ that occurred in the macaque did not occur in the gyrencephalic ferret, suggesting that this cellular behavior is not a prerequisite for the development of gyrencephalic cortex. Second, similar to gyrencephalic ferret and macaque, lissencephalic rat cortex possesses a diffuse outer band of Tbr2+ cells during late stages of cortical development.

### The distribution of Pax6-expressing cells is similar in rats and ferrets, but different in macaques

Pax6 is expressed by VZ precursor cells in the dorsal cortex, and recent work has described Pax6+ cells in the SVZ of primates and ferrets [10], [11]. We quantified the distribution of Pax6+ cells in the developing cortex during neurogenesis in each species. We quantified the number of Pax6+ cells in 200 µm wide radial bins of somatosensory cortex and compared Pax6 expression between species following the method described above. Qualitative assessment of Pax6 staining in each species revealed a similar pattern. Pax6 staining initially appeared in a dense band in the VZ, and as development proceeded an additional diffuse band of Pax6+ cells appeared in the SVZ. As with Tbr2 expression, we found more similarities in the distribution pattern of Pax6+ cells in the developing cortex of lissencephalic rat and gyrencephalic ferret, than we did between gyrencephalic ferret and macaque cortices (see Table 6 for number of Pax6+ cells analyzed).

**Figure 9:**
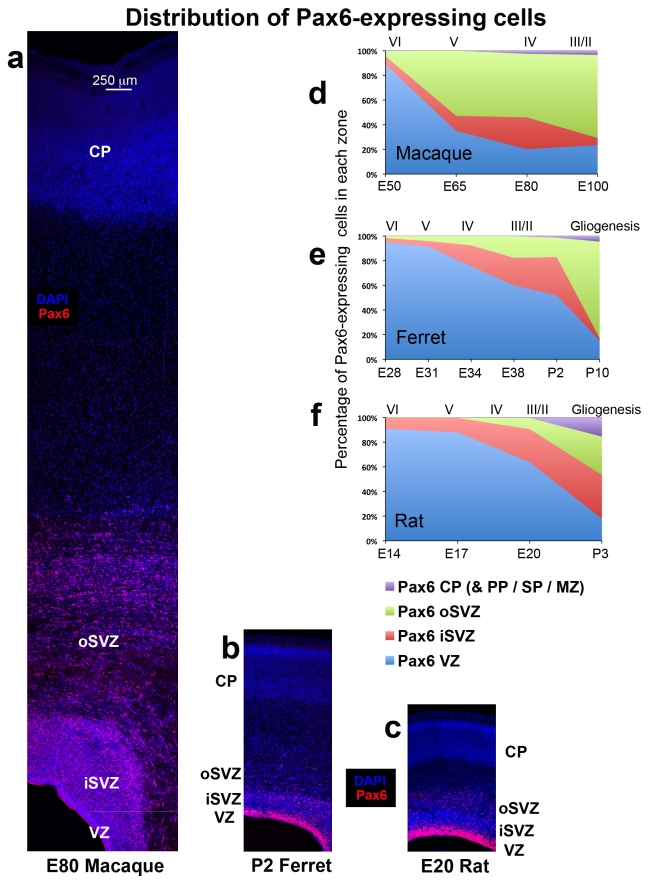
The distribution of Pax6+ cells during cortical development is similar in ferrets and rats, but different in macaques. (**a–c**) Coronal sections of somatosensory cortex from E80 macaque, P2 ferret and E20 rat immunostained for Pax6 (red) and counterstained with DAPI (blue). Images are displayed at the same scale. In each species a dense inner band of Pax6+ cells was colocalized with the ventricular zone (VZ), and a diffuse band of Pax6-expressing cells extended outward through the inner subventricular zone (iSVZ) and the outer SVZ (oSVZ). The iSVZ and oSVZ were identified based on the pattern of DAPI staining as described above. (**d–f**) Graphs showing changes in the distribution of Pax6+ cells during development of the somatosensory cortex. The stage of development is shown at the bottom of the graphs, and the approximate cortical layer generated at each stage of development is indicated along the top of each graph. (**d**) In macaque the distribution of Pax6+ cells rapidly shifted to the oSVZ. At E50 nearly 90% of Pax6+ cells were located in the VZ. But by E65 during production of layer 5 neurons [20], the majority of Pax6+ cells (60%) were located in the oSVZ. (**e**) The distribution of Pax6+ cells in the gyrencephalic ferret shifted to the oSVZ much more slowly than in macaque. At P2 during genesis of layer 2 neurons [19], 52% of Pax6+ cells remained in the VZ. (**f**) The distribution of Pax6+ cells in the lissencephalic rat was similar to that of the gyrencephalic ferret. At the end of neurogenesis on E20 during production of layer 2 neurons [21], the majority of Pax6+ cells (64%) were still located in the VZ. Legend indicates histological zones: VZ: blue; iSVZ: red; oSVZ: green. Cortical plate (CP)/preplate (PP)/subplate (SP)/marginal zone (MZ): purple. Scale bar in (**a**) applies to (**a–c**).

At E50 in the macaque 89% of Pax6+ cells were located in the VZ. However, the distribution of Pax6+ cells in macaque quickly shifted away from the ventricle during early stages of development. By E65, during production of neurons for layers 5 and 6, only 29% of Pax6+ cells remained in the VZ while approximately 67% of Pax6+ cells had shifted to the iSVZ and oSVZ, with the majority of Pax6+ cells (57%) located in the oSVZ. At E80 the percentage of Pax6+ cells in the macaque VZ decreased to 21% while the percent in the iSVZ and oSVZ had increased to 75% (Figs. 9a & d).

At early stages of cortical development in rats and ferrets the distribution of Pax6+ cells was similar to that in macaques with the majority of Pax6+ cells located in the VZ. However, the majority of Pax6+ cells remained in the VZ throughout neurogenesis in rat and ferret. At the beginning of neurogenesis, over 90% of all Pax6+ cells were located in the VZ in both species. At the end of cortical neurogenesis when layer 2 neurons were being produced, 64% of Pax6+ cells were still located in the VZ of the E20 rat somatosensory cortex (Figs. 9b & e) and 52% of Pax6+ cells remained in the VZ of the P2 ferret somatosensory cortex (Figs. 9c & f). In both rat and ferret the proportion of Pax6+ cells in the VZ dropped rapidly after neurogenesis was complete.

**Figure 10:**
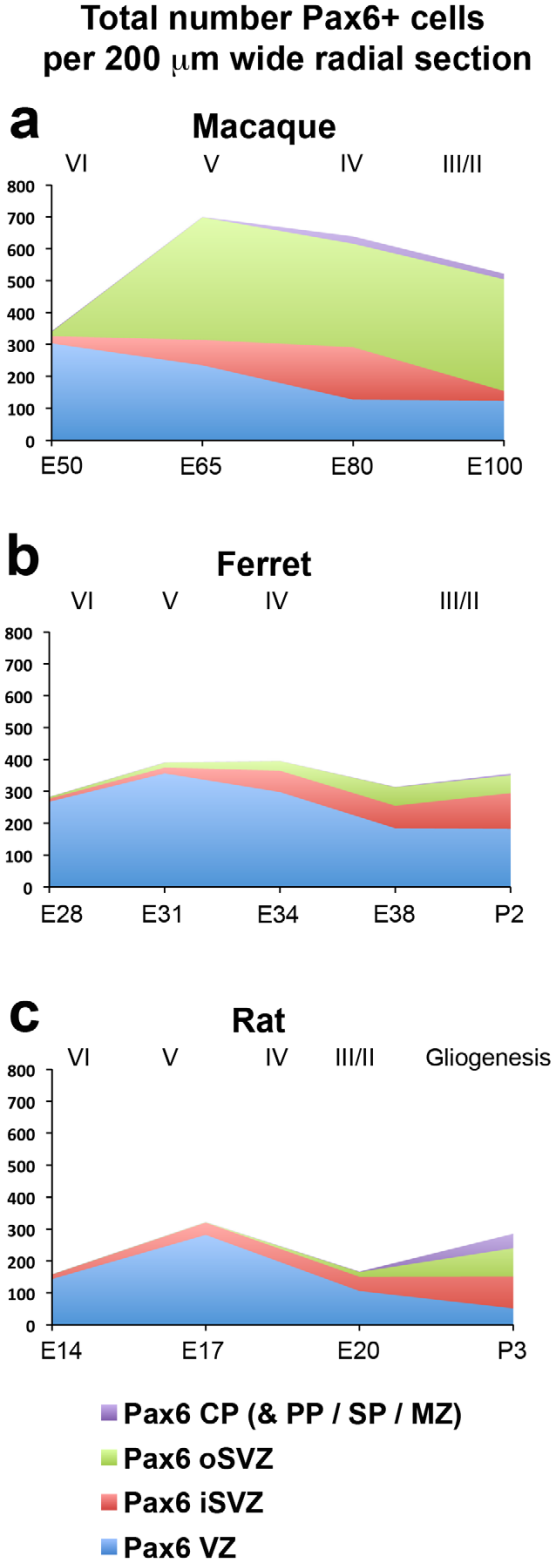
Graphs showing the total number of Pax6+ cells in the ventricular zone (VZ), inner subventricular zone (iSVZ), outer SVZ (oSVZ), and cortical plate (CP) within a 200 µm wide radial unit of macaque, ferret and rat somatosensory cortex. (**a–c**) There was a similar number of Pax6+ cells in the iSVZ of each species, but macaque had a much larger number of Pax6+ cells in the oSVZ. The stage of development is shown at the bottom of each graph, and the approximate cortical layer generated at each stage of development is indicated along the top of each graph. Legend indicates histological zones: VZ: blue; iSVZ: red; oSVZ: green; CP: purple. PP, preplate; SP, subplate; MZ, marginal zone.

We compared the total number of Pax6+ cells in each lamina of the developing cortex within 200 µm wide radial bins. This analysis showed that there were similar numbers of Pax6+ cells in the VZ and in the iSVZ per 200 µm wide bin in each species. However, macaque possessed a far greater number of Pax6-expressing cells in the oSVZ (Fig. 10, Table 7).

**Table 6:** Distribution of Pax6+ cells during neocortical development.

Macaque age	Total Pax6+ cells	Radial units	VZ	iSVZ	oSVZ	CP (incl. PP/SP/MZ)
**E50**	1023	3	89% (910/1023)	6.7% (68/1023)	4% (42/1023)	0.3% (3/1023)
**E65**	2098	3	33.9% (704/2098)	11% (239/2098)	55% (1154/2098)	0.1% (1/2098)
**E80**	1915	3	20% (381/1915)	26% (493/1915)	51% (974/1915)	3% (67/1915)
**E100**	1043	2	24% (247/1043)	6% (61/1043)	67% (701/1043)	3% (34/1043)

Ferret age	Total Pax6+ cells	Radial units	VZ	iSVZ	oSVZ	CP (incl. PP/SP/MZ)
**E28**	847	3	95% (803/847)	3% (29/847)	2% (15/847)	0% (0/847)
**E31**	1171	3	91% (1070/1171)	5% (54/1171)	3.9% (46/1171)	0.1% (1/1171)
**E34**	1185	3	75% (894/1185)	17% (201/1185)	8% (90/1185)	0% (0/1185)
**E38**	627	2	58.9% (369/627)	22% (141/627)	19% (116/627)	0.1% (1/627)
**P2**	1066	3	51% (547/1066)	32% (337/1066)	16% (168/1066)	1% (14/1066)
**P10**	3121	3	15% (476/3121)	2% (60/3121)	78% (2443/3121)	5% (142/3121)

Rat age	Total Pax6+ cells	Radial units	VZ	iSVZ	oSVZ	CP (incl. PP/SP/MZ)
**E14**	474	3	91% (431/474)	8.6% (41/474)	0.4% (2/431)	0% (0/474)
**E17**	3965	12	87% (3463/3965)	12% (484/3965)	1% (18/3965)	0% (0/3965)
**E20**	499	3	64% (318/499)	27% (134/499)	9% (45/499)	0.4% (2/499)
**P3**	855	3	18% (155/855)	35% (300/855)	31% (266/855)	16% (134/855)

Numbers represent the average percentage of Pax6+ cells in each compartment of the developing somatosensory cortex per radial unit, averaged across *n* radial units. Cells were identified through Pax6 immunoreactivity in a minimum of two 200 µm wide radial unit sections from each age in each species. Numbers in parentheses indicate the total number of Pax6+ cells counted in each compartment.

**Table 7:** Total number of Pax6+ cells/200 µm radial unit during neocortical development.

Macaque age	Average Pax6+ cells/200 µm bin	Radial units	VZ	iSVZ	oSVZ	CP (incl. PP/SP/MZ)
**E50**	342	3	303	23	15	1
**E65**	700	3	235	80	385	0
**E80**	638	3	127	164	325	22
**E100**	523	2	124	31	351	17

Ferret age	Total Pax6+ cells/200 µm bin	Radial units	VZ	iSVZ	oSVZ	CP (incl. PP/SP/MZ)
**E28**	283	3	268	10	5	0
**E31**	390	3	357	18	15	0
**E34**	395	3	298	67	30	0
**E38**	315	2	185	71	58	1
**P2**	355	3	182	112	56	5

Rat age	Total Pax6+ cells/200 µm bin	Radial units	VZ	iSVZ	oSVZ	CP (incl. PP/SP/MZ)
**E14**	159	3	144	14	1	0
**E17**	321	12	282	38	1	0
**E20**	167	3	106	45	15	1
**P3**	286	3	52	100	89	45

Numbers in each cell represent the average number of Pax6+ cells in each compartment of the developing somatosensory cortex per radial unit averaged across *n* radial units. Cells were identified through Pax6 immunoreactivity in a minimum of two 200 µm wide radial units from each age in each species.

These data show that the distribution of Pax6+ cells in the developing cortex of two gyrencephalic mammals is dramatically different. In macaque the distribution of Pax6+ cells shifts away from the ventricle into the oSVZ at an early stage of neurogenesis, but the majority of Pax6+ cells remain in the VZ of the gyrencephalic ferret and in the lissencephalic rat throughout neurogenesis. This finding supports the idea that the shift of Pax6+ cells to the oSVZ is not a prerequisite for the development of gyrencephalic cortex.

### The distribution of Tbr2+ mitoses shifts to the oSVZ during early stages of cortical development in macaque, but not in ferret or rat

We next investigated where Pax6+ or Tbr2+ cells undergo division during cortical development. For this purpose we needed a marker to unequivocally identify all cortical mitoses. We previously used the anti-phosphorylated vimentin antibody (4A4, [24]) in rat [25], and human [26], and found that it labels all mitoses in the proliferative zones of the prenatal cerebral cortex [25]. In this study we retested whether 4A4 would reliably label all mitotic cells in the developing cerebral cortex of each species. We quantified the proportion of mitotic cells that express 4A4 during cortical neurogenesis and compared that to the proportion of mitotic cells that express phosphohistone H3 (PH3) in rat. We double immunostained sections of embryonic rat cortical tissue with 4A4 and PH3 antibodies and counterstained the tissue with DAPI to label all nuclei. We identified mitoses based on the pattern of DAPI-labeled chromatin and then determined whether they expressed 4A4 or PH3. Using this straightforward approach one can readily distinguish interphase cells with dispersed chromatin from M-phase cells in prophase, metaphase, anaphase and telophase, which have condensed chromatin. We included all cells in prophase through telophase for analysis. We analyzed over 100 mitoses in the embryonic rat at E15, E16, E17 and E18, including both surface and non-surface dividing cells. All mitoses in rat cortex were strongly labeled with 4A4, but only ∼70% were labeled with the PH3 antibody. The lower proportion of PH3+ mitoses most likely reflects the lack of PH3 labeling in telophase cells [26]. We quantified the proportion of ferret and macaque cortical mitoses that express 4A4 and found that 100% of surface and non-surface mitoses were 4A4+ in both species throughout the period of neurogenesis. Since 4A4 labeling is apparent through a broader portion of M-phase it labels a larger cell population, and since 4A4 more clearly labels the somal outline of mitotic cells we used 4A4 to label cortical precursor cells for analysis of protein and transcription factor expression by precursor cells in each species (see Table 8 for number of 4A4+ precursor cells analyzed).

**Table 8:** Proportion of mitotic cells that are 4A4+ in macaque, ferret and rat.

Macaque age	Percentage of total mitoses that are 4A4+	Surface mitoses	Abventricular mitoses
**E50**	100% (617/617)	100% (476/476)	100% (141/141)
**E65**	100% (796/796)	100% (358/358)	100% (438/438)
**E80**	100% (474/474)	100% (170/170)	100% (304/304)
**E100**	100% (287/287)	100% (57/57)	100% (230/230)

Ferret age	Percentage of total mitoses that are 4A4+	Surface mitoses	Abventricular mitoses
**E28**	100% (134/134)	100% (93/93)	100% (41/41)
**E31**	100% (243/243)	100% (173/173)	100% (70/70)
**E34**	100% (213/213)	100% (123/123)	100% (90/90)
**E38**	100% (172/172)	100% (68/68)	100% (104/104)
**P2**	100% (238/238)	100% (65/65)	100% (173/173)
**P10**	100% (147/147)	100% (27/27)	100% (120/120)

Rat age	Percentage of total mitoses that are 4A4+	Surface mitoses	Abventricular mitoses
**E14**	100% (163/163)	100% (127/127)	100% (36/36)
**E17**	100% (146/146)	100% (87/87)	100% (59/59)
**E20**	100% (169/169)	100% (67/67)	100% (102/102)
**P3**	100% (135/135)	100% (12/12)	100% (123/123)

Cells were identified by condensed chromatin using DAPI staining. Numbers in parentheses indicate the total number of mitotic cells counted at each age in each species.

We observed tangential and ventricular oriented 4A4+ mitotic cells in the SVZ of each species, as we previously noted in the embryonic mouse [27]. These 4A4+ mitotic cells were Pax6+ and many had a single process up to 100 µm long that resembled the leading process of a migrating neuron, suggesting that some precursor cells actively migrate throughout the developing cortex (Fig. S1).

We next examined where Tbr2+ cells undergo division in the developing cortex. Sections of neocortical tissue were stained with 4A4 and Tbr2 antibodies, and counterstained with DAPI. We analyzed the distribution of Tbr2+ mitoses within a 300 µm wide radial bin of cerebral cortex stretching from the ventricle to the pial surface at several developmental stages in each species. We performed analysis through two approaches. For approach 1 we quantified the proportion of mitotic cells that expressed Tbr2 in each layer of the developing cortex (i.e.: what percentage of mitoses in the VZ express Tbr2, see Table 9 for number of cells analyzed). For approach 2 we quantified the percentage of Tbr2+ mitoses that were located in each zone of the developing cortex expressed as a proportion of the total number of Tbr2+ mitoses per radial bin (i.e.: of all Tbr2+ mitoses in the cortex, what proportion are located in the VZ, what proportion are located in the SVZ, etc., see Table 10 for number of cells analyzed). The first approach revealed that greater than 80% of mitotic cells in the dense inner band of the SVZ in rat and ferret expressed Tbr2, and that a lower percentage of mitotic cells in the diffuse outer band of the SVZ expressed Tbr2 during peak neurogenic stages (approximately 30–40%). The percentage of mitotic cells that expressed Tbr2 in the iSVZ was lower in the macaque (∼50%) than in rat or ferret (>80%), but the percentage of mitoses that expressed Tbr2 in macaque oSVZ was comparable to that in the diffuse outer band of rat and ferret (Table 9).

**Table 9:** Proportion of mitotic cells in each zone that are Tbr2+.

Macaque age	Percentage of total mitoses that are Tbr2+	VZ	iSVZ	oSVZ	CP (incl PP/SP/MZ)
**E50**	14% (47/325)	0% (0/236)	53% (47/89)	NA	NA
**E65**	24% (91/378)	0% (0/123)	56% (54/97)	23% (37/158)	NA
**E80**	35% (98/278)	6% (3/52)	44% (24/54)	43% (71/164)	0% (0/8)
**E100**	18% (44/249)	8% (2/26)	28% (8/29)	20% (34/166)	0% (0/28)

Ferret age	Percentage of total mitoses that are Tbr2+	VZ	iSVZ	oSVZ	CP (incl PP/SP/MZ)
**E31**	19% (29/153)	0% (0/114)	94% (29/31)	0% (0/8)	NA
**E34**	24% (36/152)	2% (2/100)	91% (30/33)	21% (4/19)	NA
**E38**	36% (33/91)	0% (0/31)	80% (24/30)	30% (9/30)	NA
**P2**	26% (36/136)	0% (0/31)	44% (27/61)	21% (9/43)	0% (0/1)
**P10**	19% (12/63)	0% (0/19)	0% (0/1)	34% (12/35)	0% (0/8)

Rat age	Percentage of total mitoses that are Tbr2+	VZ	iSVZ	oSVZ	CP (incl PP/SP/MZ)
**E14**	26% (29/110)	3% (2/81)	93% (27/29)	NA	NA
**E17**	39% (24/62)	0% (0/37)	100% (21/21)	75% (3/4)	NA
**E20**	41% (38/93)	3% (1/37)	85% (28/33)	43% (9/21)	0% (0/2)
**P3**	16% (11/70)	13% (1/8)	28% (9/32)	6% (1/17)	0% (0/13)

Numbers represent the proportion of mitotic cells in each compartment of the developing somatosensory cortex that are Tbr2+. Cells were identified through Tbr2 immunoreactivity in a minimum of three 200 µm radial units from each age in each species. Mitoses were identified with 4A4 immunoreactivity or by condensed chromatin in DAPI stained tissue. Numbers in parentheses indicate the number of Tbr2+ mitotic cells counted in each compartment from each age in each species.

**Table 10:** Distribution of Tbr2+ mitotic cells during neocortical development.

Macaque age	Total Tbr2+ mitoses	VZ	iSVZ	oSVZ
**E50**	19	0% (0/19)	100% (19/19)	0% (0/19)
**E65**	52	0% (0/52)	67% (35/52)	33% (17/52)
**E80**	72	3% (2/72)	21% (15/72)	76% (55/72)
**E100**	21	0% (0/21)	14% (3/21)	86% (18/21)

Ferret age	Total Tbr2+ mitoses	VZ	iSVZ	oSVZ
**E31**	29	0% (0/29)	100% (29/29)	0% (0/29)
**E34**	36	6% (2/36)	83% (30/36)	11% (4/36)
**E38**	33	0% (0/33)	73% (24/33)	27% (9/33)
**P2**	36	0% (0/36)	75% (27/36)	25% (9/36)
**P10**	12	0% (0/12)	0% (0/12)	100% (12/12)

Rat age	Total Tbr2+ mitoses	VZ	iSVZ	oSVZ
**E14**	29	7% (2/29)	93% (27/29)	0% (0/29)
**E17**	24	0% (0/24)	88% (21/24)	13% (3/24)
**E20**	38	2% (1/38)	74% (28/38)	24% (9/38)
**P3**	11	9% (1/11)	82% (9/11)	9% (1/11)

Numbers represent the proportion of Tbr2+ mitotic cells that were located in each compartment of the developing somatosensory cortex expressed as a percentage of the total number of Tbr2+ mitoses in a 200 µm wide radial unit. Cells were identified by Tbr2 immunoreactivity in a minimum of three 200 µm radial unit sections from each age in each species. Mitoses were identified with 4A4 immunoreactivity or by condensed chromatin in DAPI stained tissue. Numbers in parentheses indicate the total number of Tbr2+ mitotic cells counted in each compartment at each age.

**Figure 11:**
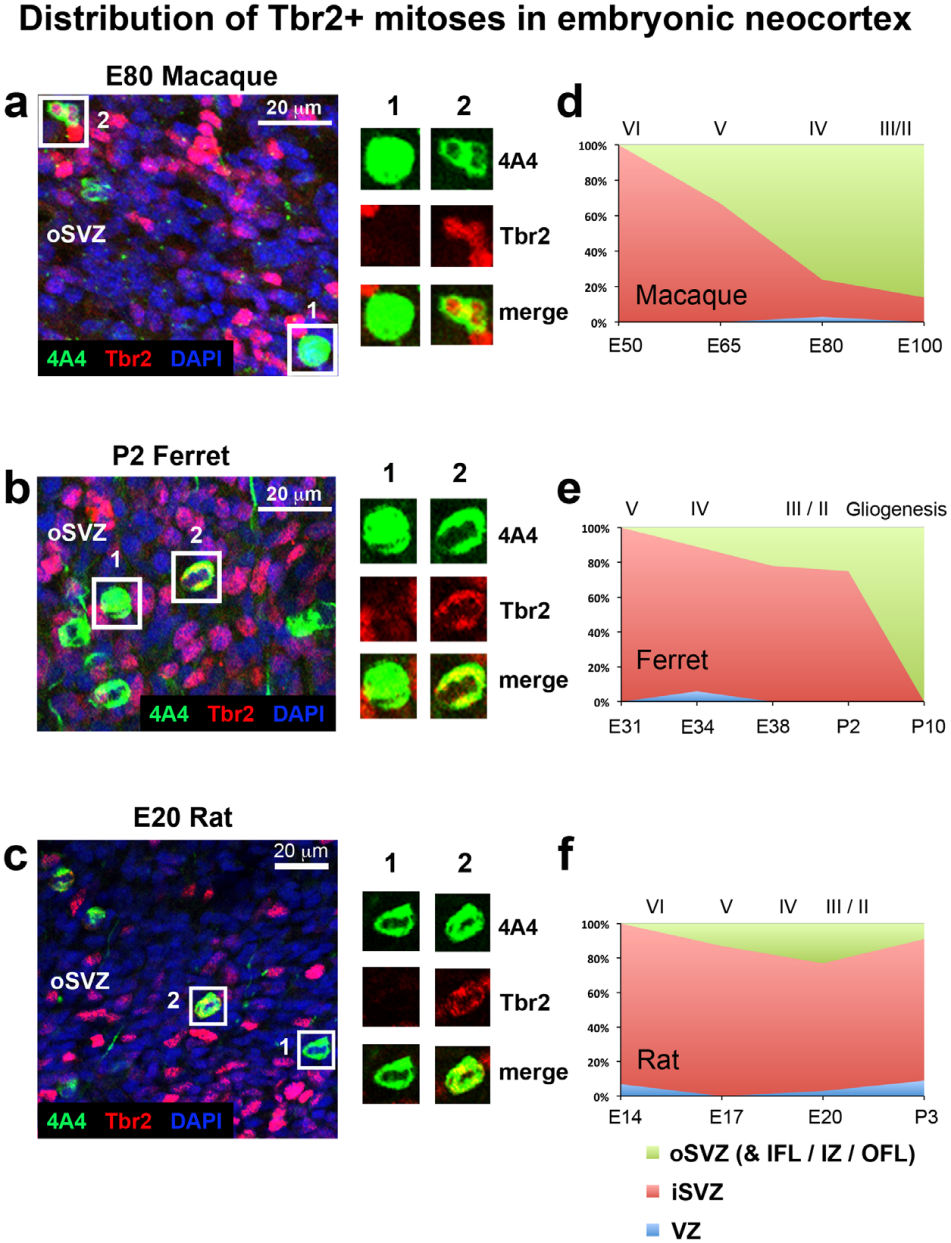
The distribution of Tbr2+ mitoses shifts to the outer subventricular zone (oSVZ) during early stages of cortical development in macaque, but not in ferret or rat. (**a–c**) Images taken from coronal sections of the E80 macaque, P2 ferret, and E20 rat oSVZ. Tissue was immunostained for 4A4 (green), Tbr2 (red) and counterstained with DAPI (blue). Boxes to the right highlight examples of 4A4+ mitotic cells that either do not express Tbr2 (#1) or that do express Tbr2 (#2). (**d–f**) Graphs showing changes in the distribution of Tbr2+ mitoses during development of the somatosensory cortex. The stage of development is shown at the bottom of the graphs, and the approximate cortical layer generated at each stage of development is indicated along the top of each graph. (**d**) At E50 in the macaque all Tbr2+ mitoses were located in the inner SVZ (iSVZ). There was a steady decrease in the proportion of Tbr2+ divisions occurring in the iSVZ. By E80 the majority of Tbr2+ mitoses (76%) had shifted to the oSVZ. (**e**) In ferret the majority of Tbr2+ mitoses were located in the iSVZ throughout neurogenesis in the somatosensory cortex. At P2, during neurogenesis of layer 2 neurons [19], 73% of Tbr2+ mitoses were still located in the iSVZ. (**f**) The pattern observed in rat was similar to that of the ferret. The majority of Tbr2+ mitoses were located in the iSVZ throughout the period of cortical neurogenesis. At P2, during production of layer 2 neurons [21], there was a similar number of Tbr2+ mitoses located in the rat iSVZ (74%) as was observed in the ferret. Legend indicates histological zones: VZ: blue; iSVZ: red; oSVZ: green.

To better understand the radial distribution of Tbr2+ mitoses across development, we adopted approach 2 and focused our analysis on the distribution of Tbr2+ mitoses in each zone expressed as a proportion of the total number of Tbr2+ mitoses in a 300 µm wide radial bin of the neocortex. In macaque we found that 100% of Tbr2 mitoses were located in the iSVZ at E50. However, the distribution of Tbr2+ mitoses quickly shifted away from the ventricle and by E80 only 21% remained in the iSVZ while 76% were located in the oSVZ (Fig. 11a & d).

In the E31 ferret, when layer 5 neurons are generated [19], 100% of Tbr2+ mitoses were located in the inner SVZ. The proportion of Tbr2+ mitoses located in the ferret inner SVZ (iSVZ) did not decrease dramatically across development as occurred in the macaque. By P2 71% of Tbr2+ mitoses in the ferret were still located in the iSVZ. We first noted Tbr2+ mitoses in the diffuse outer band of the SVZ/oSVZ at E34, during genesis of layer 4 neurons [19], when 11% of Tbr2+ mitoses were located in the oSVZ. The proportion of Tbr2+ mitoses in the ferret outer SVZ rose to 29% by P2, when layer 2 neurons are generated [19] (Fig. 11b & e).

The developmental distribution of Tbr2+ mitoses in the rat was similar to that in ferret. At E14 the SVZ had not formed as a recognizable structure, but Tbr2+ mitoses were present and 93% of the Tbr2+ mitoses were located in the upper portion of the VZ at this age. At E17 87.5% of Tbr2+ mitoses were located in the dense inner band of the SVZ and the remaining 12.5% were located in the diffuse outer band of the SVZ. At E20 the proportion of Tbr2+ mitoses located in the dense inner band was 73%, while the proportion of Tbr2+ mitoses located in the diffuse outer band of the SVZ grew to 24%. We noted that Tbr2+ mitoses were located up to 300 µm from the ventricle, which corresponds to the upper boundary of the diffuse outer band. After cortical neurogenesis at P3, the proportion of Tbr2+ mitoses located in the diffuse outer band fell to approximately 9% (Fig. 11c & f). μ

These data demonstrate that the lissencephalic rat and the gyrencephalic ferret share similar developmental distribution patterns of Tbr2+ mitoses, with the proportion of Tbr2+ mitoses located in the outer SVZ reaching a maximum of approximately 25% during neurogenesis of layer 2 cells. In contrast, a much larger proportion of Tbr2+ mitoses (>85%) were located in the macaque oSVZ during neurogenesis of layer 2 neurons. These data are consistent with the idea that the shift in the distribution of Tbr2+ mitoses to the oSVZ that occurs in macaque is not a prerequisite for the development of gyrencephalic cortex since it does not occur in ferret.

### The distribution of Pax6+ mitoses shifts to the oSVZ during early stages of cortical development in macaque, but not in ferret or rat

We next examined where Pax6-expressing cells undergo division during cortical development. We stained cortical tissue with 4A4 and Pax6 antibodies and counterstained with DAPI (see Tables 11 and 12 for the number of Pax6+ mitotic cells analyzed). We found that Pax6 was expressed by 100% of surface dividing precursor cells and by the overwhelming majority of all mitotic cells undergoing division away from the ventricle in each species.

**Table 11:** Proportion of mitotic cells in each zone that are Pax6+.

Macaque age	Proportion of total mitoses that are Pax6+	VZ	iSVZ	oSVZ	CP (incl PP/SP/MZ)
**E50**	95% (295/309)	99% (250/251)	78% (45/58)	NA	NA
**E65**	94% (238/253)	98% (58/59)	93% (79/85)	93% (101/109)	NA
**E80**	88% (235/268)	100% (40/40)	98% (40/41)	87% (154/178)	11% (1/9)
**E100**	86% (77/90)	100% (8/8)	100% (5/5)	93% (64/69)	0% (0/8)

Ferret age	Proportion of total mitoses that are Pax6+	VZ	iSVZ	oSVZ	CP (incl PP/SP/MZ)
**E28**	96% (128/134)	99% (92/93)	87% (34/39)	100% (2/2)	NA
**E31**	99% (89/90)	100% (59/59)	96% (26/27)	100% (4/4)	NA
**E34**	96% (99/103)	100% (46/46)	98% (41/42)	80% (12/15)	NA
**E38**	95% (77/81)	100% (37/37)	86% (18/21)	95% (20/21)	100% (2/2)
**P2**	96% (99/103)	100% (34/34)	100% (41/41)	92% (24/26)	0% (0/2)
**P10**	86% (72/84)	100% (8/8)	100% (4/4)	89% (54/61)	55% (6/11)

Rat age	Proportion of total mitoses that are Pax6+	VZ	iSVZ	oSVZ	CP (incl PP/SP/MZ)
**E14**	100% (53/53)	100% (46/46)	100% (7/7)	NA	NA
**E17**	98% (80/82)	100% (50/50)	91% (20/22)	100% (10/10)	NA
**E20**	97% (74/76)	100% (30/30)	100% (34/34)	91% (10/11)	0% (0/1)
**P3**	94% (61/65)	100% (4/4)	97% (28/29)	91% (21/23)	89% (8/9)

Numbers represent the proportion of mitotic cells in each compartment of the developing somatosensory cortex that are Pax6+. Cells were identified by Pax6 immunoreactivity in a minimum of three 200 µm radial units from each age in each species. Mitoses were identified with 4A4 immunoreactivity or by condensed chromatin in DAPI stained tissue. Numbers in parentheses indicate the number of Pax6+ mitotic cells and total mitotic cells counted in each compartment from each age in each species.

**Table 12:** Distribution of Pax6+ mitotic cells during neocortical development.

Macaque age	Total Pax6+ mitoses	VZ	iSVZ	oSVZ	CP (incl. PP/SP/MZ)
**E50**	99	80% (79/99)	20% (20/99)	0% (0/99)	0% (0/99)
**E65**	123	47% (58/123)	33% (40/123)	20% (25/123)	0% (0/123)
**E80**	132	10% (13/132)	28% (37/132)	61% (81/132)	1% (1/132)
**E100**	39	8% (3/39)	13% (5/39)	79% (31/39)	0% (0/39)

Ferret age	Total Pax6+ mitoses	VZ	iSVZ	oSVZ	CP (incl. PP/SP/MZ)
**E28**	128	72% (92/128)	27% (34/128)	1% (2/128)	0% (0/128)
**E31**	89	66% (59/89)	29% (26/89)	5% (4/89)	0% (0/89)
**E34**	99	47% (46/99)	41% (41/99)	12% (12/99)	0% (0/99)
**E38**	77	48% (37/77)	23% (18/77)	26% (20/77)	3% (2/77)
**P2**	99	34% (34/99)	42% (41/99)	24% (24/99)	0% (0/99)

Rat age	Total Pax6+ mitoses	VZ	iSVZ	oSVZ	CP (incl. PP/SP/MZ)
**E14**	53	87% (46/53)	13% (7/53)	0% (0/53)	0% (0/53)
**E17**	80	63% (50/80)	25% (20/80)	12% (10/80)	0% (0/80)
**E20**	74	41% (30/74)	46% (34/74)	13% (10/74)	0% (0/74)
**P3**	61	7% (4/61)	46% (28/61)	34% (21/61)	13% (8/61)

Numbers represent the proportion of Pax6+ mitotic cells in each compartment of the developing somatosensory cortex in each species. Cells were identified by Pax6 immunoreactivity in a minimum of three 200 µm radial unit sections from each age in each species. Mitoses were identified by 4A4 immunoreactivity and/or condensed chromatin in DAPI stained tissue. Numbers in parentheses indicate the total number of Pax6+ mitotic cells counted in each compartment from each age.

**Figure 12:**
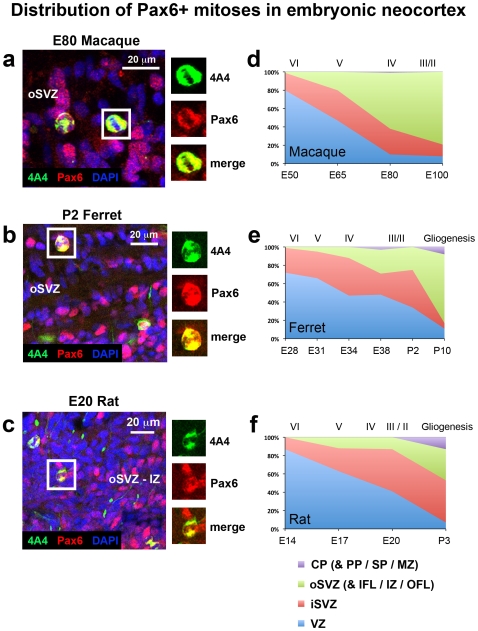
The distribution of Pax6+ mitoses shifts from the ventricular zone (VZ) to the subventricular zone (oSVZ) during early stages of cortical development in macaque, but not in ferret or rat. (**a–c**) Images taken from coronal sections of the E80 macaque, P2 ferret and E20 rat oSVZ. Tissue was immunostained for 4A4 (green), Pax6 (red) and counterstained with DAPI (blue). Boxes to the right highlight examples of 4A4+ mitotic cells that express Pax6. (**d–f**) Graphs showing changes in the distribution of Pax6+ mitoses during development of the somatosensory cortex. The stage of development is shown at the bottom of the graphs, and the approximate cortical layer generated at each stage of development is indicated along the top of each graph. (**d**) At E50 in the macaque nearly 90% of Pax6+ mitoses were located in the VZ. There was a steady shift in the distribution of mitoses to the outer SVZ (oSVZ). By E80 the majority of Pax6+ mitoses (61%) were located in the oSVZ. (**e**) In ferret the largest proportion of Pax6+ mitoses were located in the VZ until late stages of neurogenesis. At E38, during neurogenesis of layer 2 neurons [19], 48% of Pax6+ mitoses were still located in the VZ, 23% were located in the iSVZ and 26% in the oSVZ. (**f**) A similar pattern was observed in the rat. The majority of Pax6+ mitoses were located in the VZ until E20. At E20 40% of Pax6+ mitoses were located in the VZ, 45% were in the iSVZ and 13% in the oSVZ. Legend indicates histological zones: VZ: blue; iSVZ: red; oSVZ: green; CP: purple. PP, preplate; SP, subplate; MZ, marginal zone.

In the macaque 100% of surface mitoses were Pax6+ at each age and a smaller proportion of mitotic cells expressed Pax6 away from the ventricle, particularly at later stages of development. We examined the distribution of Pax6+ mitoses in macaque and found that the distribution of Pax6+ mitoses shifted away from the ventricle as development proceeded. At E50 80% of Pax6+ mitoses were located in the VZ, but by E65, during production of layer 5 and 6 neurons [20], the majority (53%) were located in the iSVZ and oSVZ. The shift in the distribution of Pax6+ mitoses from the VZ to the SVZ continued through development. By E80 the proportion of Pax6+ mitoses in the SVZ was 79%, and at E100 93% of Pax6+ mitoses were located in the SVZ, with the majority of Pax6+ mitotic precursor cells (79%) located in the oSVZ (Fig. 12a & d).

In the ferret we found that 90 to 100% of all mitoses expressed Pax6 in each lamina of the developing cortex. Few 4A4+ cells were found beyond the oSVZ, but most of these mitoses were also Pax6+. We noted a steady increase in the proportion of Pax6+ divisions that occurred away from the ventricle in the iSVZ and oSVZ as development proceeded. However, in comparison to macaque, the shift in the distribution of Pax6+ mitoses away from the ventricle in the ferret occurred more slowly, at a later stage of cortical development and was not as pronounced. At E34, during production of layer 4 neurons [19], the majority of Pax6+ mitoses (53%) were located in the iSVZ and oSVZ. At P2, near the end of neurogenesis in ferret somatosensory cortex [19], the proportion of Pax6+ mitoses in the iSVZ and oSVZ reached 66% (Fig. 12b & e).

We next examined the distribution of Pax6+ mitoses in the developing rat. As in the ferret we noted a steady increase in the proportion of Pax6+ mitoses that occurred away from the ventricle in the dense inner band and diffuse outer band of the SVZ as development proceeded. At E17 only 37% of Pax6+ mitoses were located in the dense inner and diffuse outer subdivisions of the SVZ, but near the end of neurogenesis at E20 this proportion rose to 58% (Fig. 12c & f).

### Mitotic precursor cells coexpress Tbr2 and Pax6 in the oSVZ

**Figure 13:**
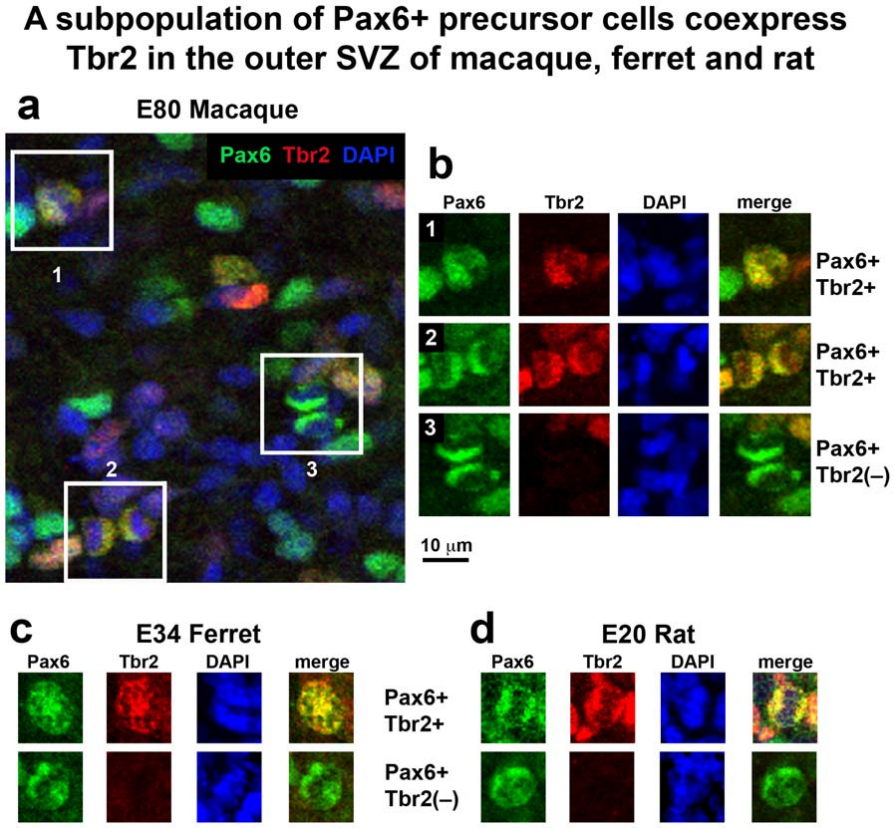
A subpopulation of Pax6+ mitotic cells coexpress Tbr2 in the outer subventricular zone (oSVZ) of macaque, ferret, and rat dorsal somatosensory cortex. (**a**) Image from E80 macaque oSVZ immunostained for Pax6 (green) and Tbr2 (red), and counterstained with DAPI (blue). Boxes highlight examples of Pax6+ mitoses. (**b**) Examples of Pax6+ mitotic cells from insets in (**a**) demonstrating two examples of Pax6+ mitoses that coexpress Tbr2 (1 and 2) and one example that does not (3). (**c**) E34 ferret oSVZ. An example of a Pax6+ mitosis that coexpresses Tbr2 and a second example that does not. (**d**) E20 rat oSVZ. An example of a Pax6+ mitosis that coexpresses Tbr2 and a second example that does not. In all three species most Tbr2+ mitotic cells express Pax6, although in most cases the level of Pax6 expression is low in Tbr2+ cells. There are significant numbers of Pax6+ mitotic cells in the oSVZ of each species that do not express Tbr2 (see Table 13). Scale bar applies to all images.

We found that in rat, ferret and macaque most Tbr2+ mitoses also expressed Pax6 at either weak or strong levels. In the macaque 90% of abventricular Tbr2 mitoses were also Pax6+; in the ferret 95% of abventricular Tbr2+ mitoses were also Pax6+; and in the rat 95% of Tbr2+ mitoses were also Pax6+ (Fig. 13, Table 13). We noted that in most cases the level of Pax6 expression was lower in Tbr2-positive mitoses than the level of Pax6 expression in Tbr2-negative mitoses. A small subset of Tbr2+ mitoses also expressed strong levels of Pax6. We also examined the proportion of Pax6+ mitoses that expressed Tbr2 and found that it was much lower. Approximately 50% of Pax6+ precursor cells expressed Tbr2 in macaque and in rat, while roughly one third of Pax6+ precursor cells expressed Tbr2 in the ferret (Table 13). These data indicate that there are at least two distinct populations of Pax6+ precursor cells in the developing brain based on Tbr2 expression. Previous studies that examined co-expression of the Pax6 and Tbr2 transcription factors reported that 85% of Pax6+ interphase cells in the SVZ expressed Tbr2 in the E14.5 mouse [28], or that 11% of cells in the VZ, SVZ and IZ of E14.5 mouse coexpressed Pax6 and Tbr2 [7], or that there was no expression of Pax6 by precursor cells outside of the VZ in the E16.5 mouse [6]. Differences in co-expression studies may result from different antibodies or staining protocols, from examination of different developmental stages, or because our analysis focused exclusively on mitotic cells. Of note, our data shows that Tbr2+ mitoses co-express Pax6 in the diffuse outer band of the SVZ in lissencephalic rat, and in the oSVZ of the gyrencephalic ferret and macaque. In addition, we also find Pax6+ mitoses that do not co-express Tbr2 in the diffuse outer band of the rat SVZ, and in the oSVZ of ferret and macaque. These data provide further evidence that the outer portion of the SVZ comprises similar cell types in rat, ferret and macaque.

**Table 13:** Coexpression of Pax6 and Tbr2 in mitotic cells.

Macaque age	Total Pax6+ mitoses	Total Tbr2+ mitoses	Total Pax6+ Tbr2+ mitoses	Percentage of Pax6+ mitoses that are Tbr2+	Percentage of Tbr2+ mitoses that are Pax6+
**E50**	176	47	30	17% (30/176)	64% (30/47)
**E65**	244	122	120	49% (120/244)	98% (120/122)
**E80**	72	37	37	51% (37/72)	100% (37/37)
**E100**	38	20	17	45% (17/38)	85% (17/20)

Ferret age	Total Pax6+ mitoses	Total Tbr2+ mitoses	Total Pax6+ Tbr2+ mitoses	Percentage of Pax6+ mitoses that are Tbr2+	Percentage of Tbr2+ mitoses that are Pax6+
**E28**	55	22	19	35% (19/55)	86% (19/22)
**E31**	52	8	8	15% (8/52)	100% (8/8)
**E34**	115	36	34	30% (34/115)	94% (34/36)
**P2**	66	17	17	26% (17/66)	100% (17/17)
**P10**	26	6	6	23% (6/26)	100% (6/6)

Rat age	Total Pax6+ mitoses	Total Tbr2+ mitoses	Total Pax6+ Tbr2+ mitoses	Percentage of Pax6+ mitoses that are Tbr2+	Percentage of Tbr2+ mitoses that are Pax6+
**E20**	108	58	55	51% (55/108)	95% (55/58)

Numbers represent the coexpression of Pax6 and Tbr2 by mitotic cells in the developing somatosensory cortex in each species. Cells were identified by Pax6 and/or Tbr2 immunoreactivity in a minimum of three 200 µm radial unit sections from each age in each species. Mitoses were identified by condensed chromatin in DAPI stained tissue. Numbers in parentheses indicate the total number of mitotic cells of each type (Pax6 or Tbr2) that coexpress both proteins.

### tRG cells are present in embryonic rat neocortex

During embryonic cortical development in rats and mice, radial glial cells undergo divisions that produce translocating radial glial (tRG) daughter cells that inherit the pial process and translocate away from the VZ toward the cortical plate [4], [16]. The tRG cells in rat share features with radial glial cells including similar electrophysiological membrane properties, as well as vimentin and nestin expression [4], [5]. The tRG cells remain mitotic as they translocate toward the cortical plate [4], [5], [16]. The daughter cells produced by dividing tRG may either be non-neuronal, based on electrophysiological recordings [5], or in the neuronal lineage, based on expression of the Hu protein [16], or NeuN [17]. Studies have shown that Pax6+ tRG cells in the human oSVZ produce Tbr2+ intermediate progenitor cells [10].

**Figure 14:**
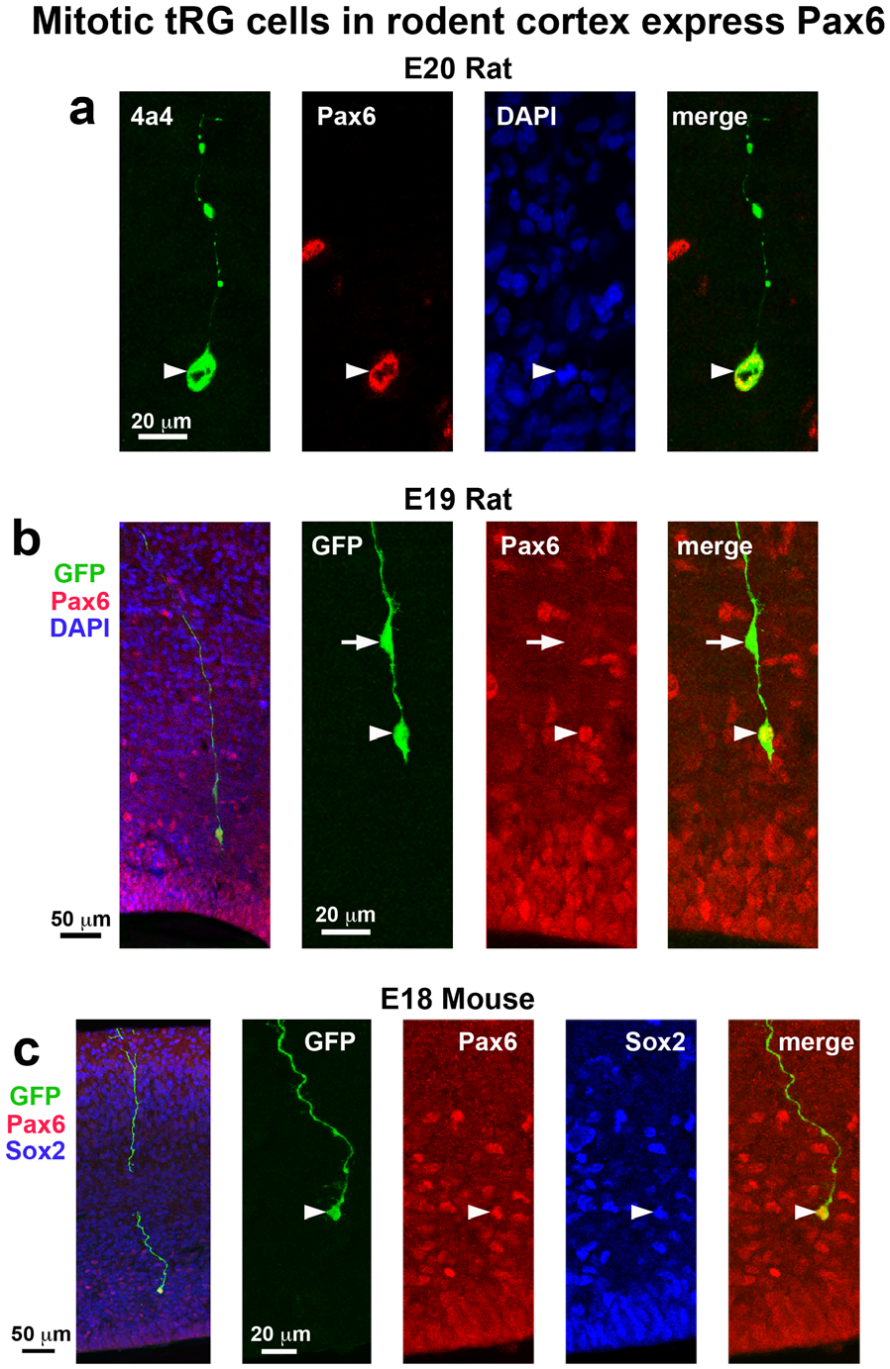
Mitotic translocating radial glial (tRG) cells in rodent cortex express Pax6. (**a**) Example of a mitotic tRG cell labeled with 4A4 (green). The pial fiber of the dividing cell is seen coursing to the pial surface. All mitotic tRG cells coexpressed Pax6 (red). DAPI stained nuclei are shown (blue). (**b**) An example of a GFP+ tRG cell (green) labeled through in utero retroviral injection at E16. The tRG cell (arrowhead) expresses Pax6 (red). A daughter cell can be seen migrating along the pial fiber (arrow). All tRG cells labeled with retroviral injections expressed Pax6. (**c**) tRG cells labeled in the E18 mouse through in utero electroporation with a GFP expressing plasmid (green) expressed both Pax6 (red) and Sox2 (blue). Left panel shows a low magnification merged image of the tRG cell. Middle images show high magnification individual images of GFP (green), Pax6 (red) and Sox2 (blue). Right image shows high magnification merged image of GFP (green), Pax6 (red) and Sox2 (blue). All GFP+ cells with tRG morphology in mouse were Pax6+ and Sox2+.

The 4A4 antibody labels mitotic RG with intact pial fibers in the embryonic rat cerebral cortex [25]. We found that 4A4 also labels numerous cells in the outer SVZ of each species that possessed the characteristic morphology of tRG cells, including a long pial process extending toward the cortical plate. We counterstained tissue with 4A4 and Pax6 antibodies and found that all 4A4+ mitotic cells with tRG morphology expressed Pax6 (Fig. 14a). To more closely examine the morphology of apparent tRG cells, we performed retroviral injections in the E16 to E20 rat neocortex and allowed embryos to continue developing in utero several days after the retroviral injections, at which point embryos were perfused, sectioned and immunostained with Pax6 antibodies. We found that all retrovirally labeled GFP+ cells with tRG morphology in the rat neocortex expressed Pax6 (Fig. 14b). Similar experiments performed in developing mice produced the same result. In addition we found that the murine Pax6+ tRG cells also expressed the precursor cell marker Sox2 (Fig. 14c).

**Figure 15:**
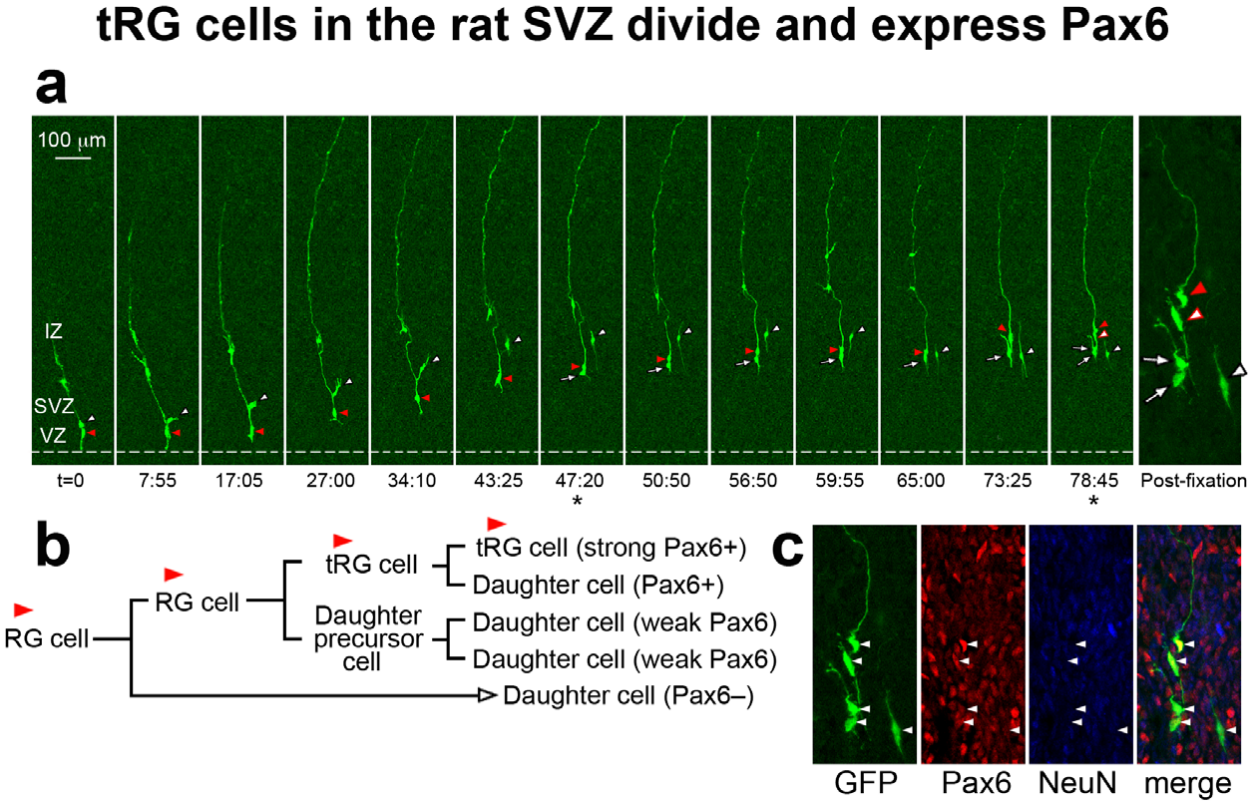
Translocating RG (tRG) cells in the embryonic rat oSVZ divide and express Pax6. (**a**) Time lapse recording of a GFP-labeled tRG cell in the rat somatosensory cortex after an E16 retroviral injection. At the start of the experiment the labeled cell (red arrowhead) has RG morphology including a ventricular process and a pial process. The first timepoints of this movie focused on the RG cell body in the VZ and did not capture the pial process. An RG daughter cell (white arrowhead) is in contact with the pial process. The RG cell lost contact with the ventricle at t = 17 h, acquired tRG morphology, and began translocating toward the cortical plate. The first daughter cell (white arrowhead) remained in close proximity to its parent tRG cell for the duration of the experiment and did not divide. The tRG cell divided again at approximately t = 47 h (asterisk), producing a self-renewed tRG cell (red arrowhead) and a second daughter cell (white arrow). The tRG cell continued to translocate and divided once again at approximately t = 78 h, producing a third daughter cell (white arrowhead with red border). In addition, the second daughter cell also divided at approximately t = 78 h, producing two daughter cells with similar morphology. After an additional two hours the section was fixed in 4% PFA, sectioned on a cryostat and immunostained for Pax6 and NeuN. The clonal cells are shown on the far right image at higher magnification after fixation. (**b**) Lineage tree depicting the progeny of the RG cell in this time-lapse recording. (**c**) Immunostaining shows that the tRG cell expresses Pax6. The first daughter cell did not express Pax6. The daughter cells that were produced by division of the tRG second daughter cell (white arrow) expressed weak levels of Pax6. The tRG third daughter cell (white arrowhead with red border) expressed a moderate level of Pax6 that was lower in comparison to the level of Pax6 expression by the tRG mother cell. All cells in this clone were NeuN-negative. White arrowheads indicate the locations of clonal cells in each panel. This data shows that mitotically active RG cells and tRG cells maintain high levels of Pax6 expression, and suggests that non-RG daughter cells slowly downregulate Pax6 expression as they migrate toward the cortical plate. Scale bar in the right panel of (**a**) applies to all images in (**c**). VZ, ventricular zone; SVZ, subventricular zone; IZ, intermediate zone.

To determine if cells with apparent tRG morphology in the developing rat cortex were actually translocating cells, we performed retroviral injections in E16 to E20 embryos and prepared organotypic slices 24–72 hours after injections for time-lapse imaging of GFP-labeled cells in the embryonic cortex as described previously [3]. We imaged RG cells undergoing division at the surface of the ventricle and recorded the movements of daughter cells, paying special attention to the daughter cell that inherited the pial process. During later stages of embryonic rat cortical development, RG daughter cells that inherited the pial process either remained in the VZ as RG cells or acquired tRG cell morphology, detached from the lateral ventricle and translocated away from the VZ following the radial trajectory of the pial process. We noted that many of the tRG cells continued dividing as they translocated toward the cortical plate, as we have previously shown [4], [5]. After the tRG cells had migrated away from the VZ we fixed and immunostained the cultured slices with Pax6 antibodies. We found that rat tRG cells expressed Pax6 (Fig. 15). Mitotic RG/tRG cells exhibited strong levels of Pax6 expression and we noted that the level of Pax6 expression appeared to decrease over time in the daughter cells produced by radial glia and tRG cells. For example, Figure 15 shows an example of an RG cell that transitioned into a tRG cell. We followed the tRG cell and three generations of daughter cells. The first daughter cell produced (indicated with a white arrowhead in Fig. 15a) shared some characteristics with neurons: it migrated through locomotion, exhibited retrograde migratory movements toward the ventricle, did not divide and was Pax6-negative after three days of time-lapse imaging. Nonetheless, this daughter cell did not express NeuN although 78 hours had passed after it was generated (Fig. 15c). The second daughter cell produced by the tRG cell (t = 47 h, indicated with a white arrow in Fig. 15a) was a presumed intermediate progenitor cell that subsequently divided at t = 74 h, and produced two daughter cells that expressed weak levels of Pax6. The tRG cell produced a third daughter cell at the end of the time-lapse experiment (t = 78 h, indicated by a white arrow with a red border) that expressed higher levels of Pax6 than the previous generations of daughter cells, but lower levels of Pax6 expression than its tRG mother cell (Fig. 15c). These data suggest a pattern of Pax6 expression during neurogenic divisions whereby actively dividing RG and tRG cells retain high levels of Pax6 expression while the RG daughter cells slowly lose Pax6 expression as they differentiate into neurons or intermediate progenitor cells that express Tbr2. This idea is consistent with our data showing that many Tbr2+ cells express low levels of Pax6 (Fig. 13). We have previously shown that tRG daughter cells in the embryonic cortex lack the physiological membrane properties of neurons, consistent with the idea that tRG cells in the embryonic cortex can generate glial daughter cells [4], [5]. We presume that astroglial daughter cells produced during prenatal development would retain Pax6 expression. Importantly, this data confirms that tRG cells express Pax6 in the rat as they do in ferret and macaque.

### Mitotically active Pax6+ tRG Cells are present during and after cortical neurogenesis

**Figure 16:**
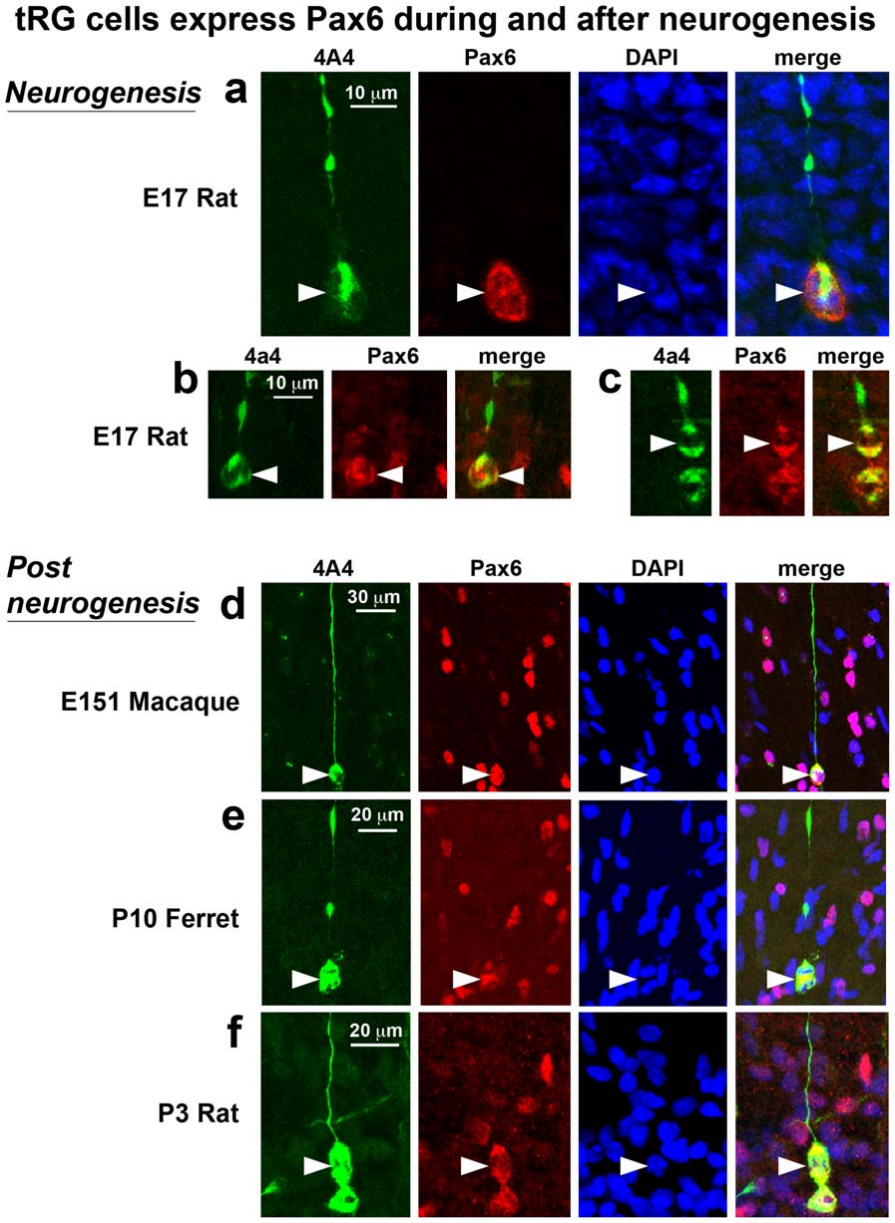
tRG cells express Pax6 during and after neurogenesis. (**a–c**) 4A4+ tRG cells (green) in the E17 rat somatosensory cortex that express Pax6 (red). Blue channel shows DAPI staining. (**d–f**) Examples of 4A4+ tRG cells (green) in post-neurogenic somatosensory cortex of macaque (**d**), ferret (**e**), and rat (**f**) that also express Pax6 (red). Blue channel shows DAPI staining. Scale bar in left panels applies to all panels in each set.

**Figure 17:**
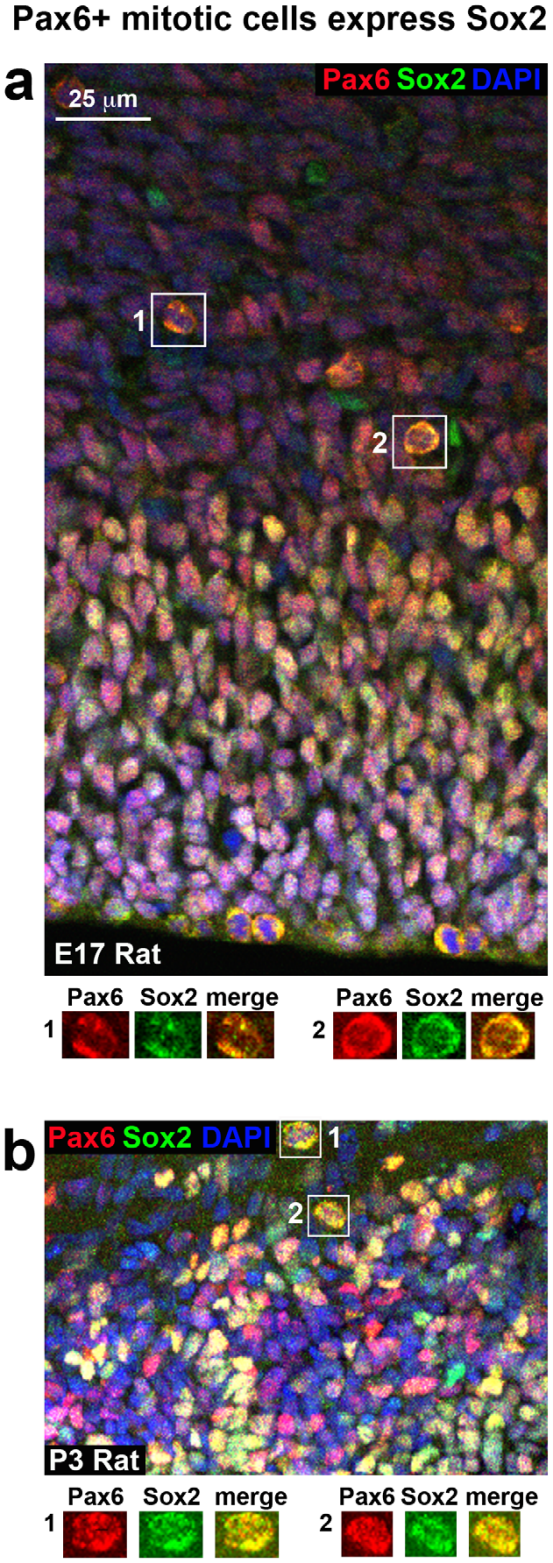
Most mitotic Pax6+ cells express Sox2 during and after neurogenesis. (**a**) Coronal section from E17 rat somatosensory cortex immunostained for Pax6 (red) and Sox2 (green) and counterstained with DAPI (blue). Boxes (1 and 2) show examples of Pax6+ mitotic cells that also express Sox2. (**b**) Coronal section from P3 rat somatosensory cortex immunostained for Pax6 (red) and Sox2 (green) and counterstained with DAPI (blue). Boxes (1 and 2) show examples of Pax6+ mitotic cells that also express Sox2. There was greater overlap of Pax6 and Sox2 expression at E17 than at P3, but the extent of colabeling in mitotic cells was similar at each age (see Table 14). Scale bar in (**a**) applies to all images.

We found that Pax6-expressing mitotically active tRG cells in each species were present both during and after cortical neurogenesis (Fig. 16), which suggests that tRG cells may be both neurogenic and gliogenic. We double immunostained tissue with Pax6 and Sox2 antibodies to determine if we could distinguish two subtypes of tRG cells. However, we found that nearly all Pax6+ mitotic cells in the SVZ also expressed Sox2, both during and after neurogenesis (Fig. 17, Table 14). This may not be surprising since Sox2 is expressed by both neuronal and glial precursor cells in the developing dorsal telencephalon [29]. At both E17 and P3 over 95% of Pax6+ mitotic cells expressed Sox2. We next tested the possibility that the Pax6+ tRG cells in the postnatal rat (e.g.: P3 rat) generate the last compliment of superficial layer 2 neurons. We pulsed rats with two injections of BrdU (80 mg/kg) at P1, P3, and P7. Animals were sacrificed on P10 and neocortical tissue was co-immunostained with antibodies against NeuN and BrdU. P1 BrdU injections produced a very small number of BrdU+ nuclei that appeared to be weakly positive for NeuN in the medial cingulate cortex. However, no BrdU+ nuclei expressed NeuN in the dorsolateral cortex. P3 and P10 injections did not produce BrdU+/NeuN+ cells in the cerebral cortex of any animals, consistent with previous data on rat neurogenesis [21].

**Table 14:** Coexpression of Sox2 and Pax6 in mitotic cells.

Rat age	Total Sox2+ mitoses	Total Pax6+ mitoses	Total Sox2+ Pax6+ mitoses	Percentage of Sox+ mitoses that are Pax6+	Percentage of Pax6+ mitoses that are Sox2+
**E14**	26	26	26	100% (26/26)	100% (26/26)
**E17**	81	83	79	98% (79/81)	95% (79/83)
**E20**	76	89	75	99% (75/76)	84% (75/89)
**P3**	32	32	31	97% (31/32)	97% (31/32)

Numbers represent the coexpression of Sox2 and Pax6 by mitotic cells in the developing rat somatosensory cortex. Cells were identified by Sox2 and/or Pax6 immunoreactivity in a minimum of three 200 µm radial unit sections at each age. Mitoses were identified by condensed chromatin in DAPI stained tissue. Numbers in parentheses indicate the total number of mitotic cells of each type (Sox2 or Pax6) that coexpress both proteins.

These data show that tRG cells in the rodent SVZ express Pax6 as they do in ferret and macaque and that the diffuse outer band of the embryonic SVZ in rodents consists in part of Pax6+ tRG cells, as does the oSVZ of ferret and macaque (Fig. 16), and the human oSVZ [10], [11]. Furthermore, these data strongly suggest that Pax6+ tRG cells that are present in the postnatal cortex are not neurogenic.

### Olig2 expression distinguishes two distinct subtypes of tRG cells in the developing cerebral cortex

**Figure 18:**
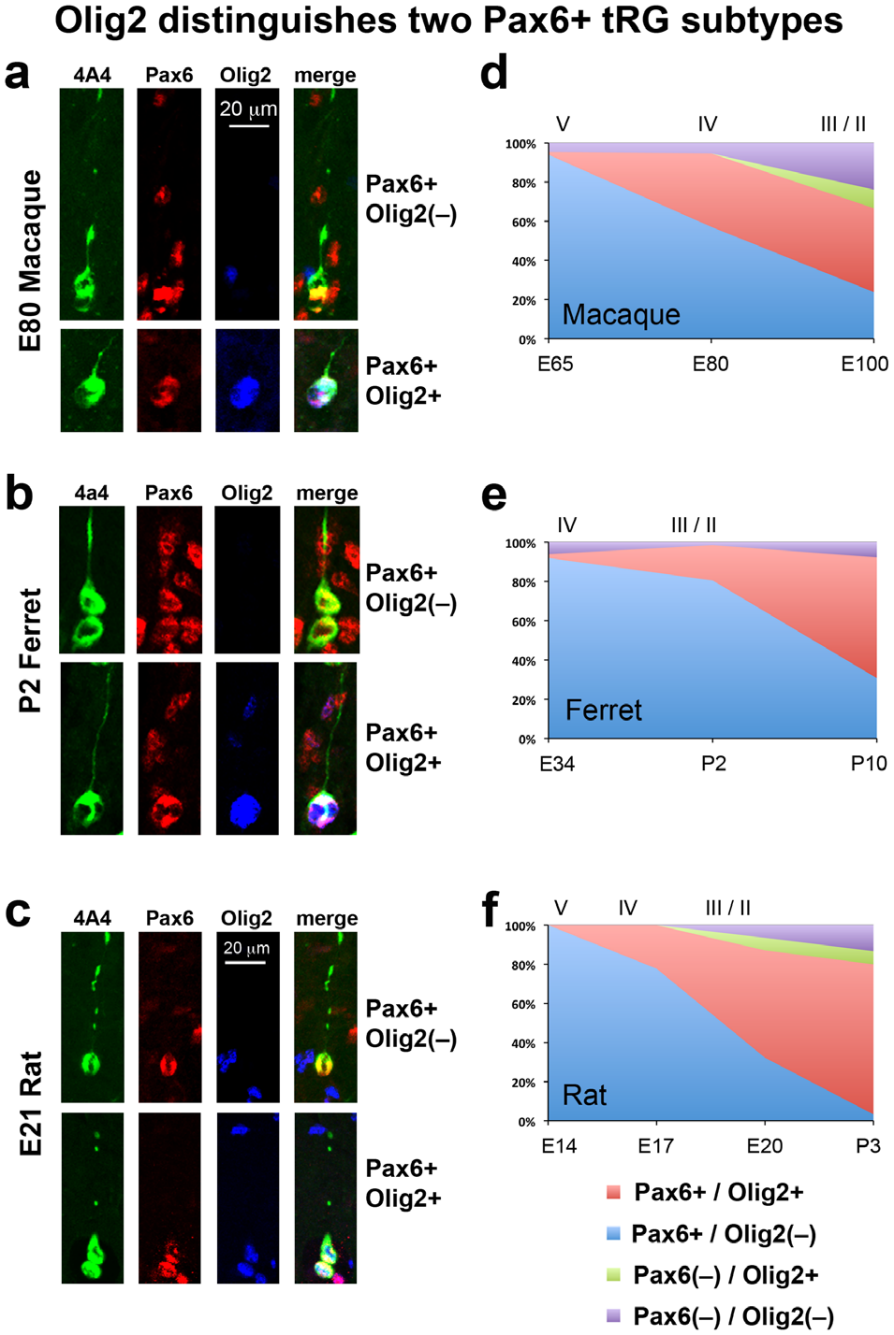
Olig2 expression distinguishes two subtypes of tRG cells in macaque, ferret and rat. (**a–c**) Examples of 4A4+ tRG cells (green) in the E80 macaque (**a**), P2 ferret (**b**), and E21 rat (**c**), that express Pax6 (red). Shown are examples of Pax6+ tRG cells that are Olig2-negative (top row of panels), or Olig2+ (lower row of panels, Olig2 immunostaining: blue). (**d–f**) Graphs showing changes in the proportion of Pax6+ tRG cells that express Olig2 during cortical development. During early stages of cortical development most Pax6+ cells were Olig2-negative in each species. The proportion of Pax6+ tRG cells that expressed Olig2 increased as development proceeded. Graphs also show Pax6(−)/Olig2+, or Pax6(−)/Olig2(−) 4A4+ cells with a pial fiber. Scale bar in (**a**) applies to (**b**).

Our data demonstrate that Pax6 expression alone is not enough to identify neurogenic precursor cells and that Pax6+ mitotic cells are not neurogenic in the postnatal cortex. Since we previously presented data showing that tRG cells can produce non-neuronal daughter cells [4], [5], we explored the possibility that a subset of Pax6+ tRG cells in the embryonic neocortex are gliogenic. Since Olig2 directs astrocyte formation in the postnatal SVZ [30], we used 4A4 immunostaining to identify mitotic tRG cells in the outer SVZ. Presumed 4A4+ tRG cells possessed a process that was directed toward the pia. We asked whether these cells expressed Pax6 and Olig2. We noted strong Olig2 expression in a subset of Pax6+ tRG cells during late stages of embryonic rat cortical development (Fig. 18a, Table 15).

**Table 15:** Pax6 and Olig2 expression by 4A4+ cells with a pial fiber (tRG cells).

Macaque age	Total tRG cells	Proportion of tRG cells that are Pax6+/Olig2(−)	Proportion of tRG cells that are Pax6+/Olig2+	Proportion of tRG cells that are Pax6(−)/Olig2+	Proportion of tRG cells that are Pax6(−)/Olig2(−)
**E65**	66	94% (62/66)	1% (1/66)	0% (0/66)	5% (3/66)
**E80**	56	57% (32/56)	38% (21/56)	0% (0/56)	5% (3/56)
**E100**	21	24% (5/21)	43% (9/21)	9% (2/21)	24% (5/21)

Ferret age	Total tRG cells	Proportion of tRG cells that are Pax6+/Olig2(−)	Proportion of tRG cells that are Pax6+/Olig2+	Proportion of tRG cells that are Pax6(−)/Olig2+	Proportion of tRG cells that are Pax6(−)/Olig2(−)
**E34**	50	92% (46/50)	2% (1/50)	0% (0/50)	6% (3/50)
**P2**	72	81% (58/72)	18% (13/72)	0% (0/72)	1% (1/72)
**P10**	26	31% (8/26)	61% (16/26)	0% (0/26)	8% (2/26)

Rat age	Total tRG cells	Proportion of tRG cells that are Pax6+/Olig2(−)	Proportion of tRG cells that are Pax6+/Olig2+	Proportion of tRG cells that are Pax6(−)/Olig2+	Proportion of tRG cells that are Pax6(−)/Olig2(−)
**E17**	18	100% (18/18)	0% (0/18)	0% (0/18)	0% (0/18)
**E19**	54	78% (42/54)	22% (12/54)	0% (0/54)	0% (0/54)
**E21**	31	33% (10/31)	55% (17/31)	6% (2/31)	6% (2/31)
**P2**	30	3% (1/30)	77% (23/30)	7% (2/30)	13% (4/30)

Numbers represent the expression of Pax6 and Olig2 by 4A4+ mitotic cells with a pial fiber (tRG cells) in the developing somatosensory cortex in each species. Cells were identified by 4A4, Pax6 and Olig2 immunoreactivity in a minimum of three 200 µm radial units from each age in each species. Numbers in parentheses indicate the total number of tRG cells of each type (Pax6 or Olig2) that coexpress both proteins.

In the developing macaque we found that the majority of mitotic Pax6+ tRG cells (94%) were Olig2 negative at E65 and that only 2% expressed Olig2. At E80 57% of mitotic Pax6+ tRG cells were Olig2 negative and 38% expressed Olig2. By E100, representing the end of macaque cortical neurogenesis [20], the proportion of mitotic Pax6+ tRG cells that were Olig2 negative was 24% and the proportion that were Olig2+ increased to 43% (Fig. 18a & d). We further examined the possibility that tRG cell subtypes exist in the developing macaque neocortex by counterstaining tissue with 4A4 and GFAP, but qualitatively found that all 4A4+ tRG cells were GFAP+.

In the developing ferret we found that the majority of mitotic Pax6+ tRG cells (92%) were Olig2 negative at E34 and only 2% expressed Olig2. At P2 81% of mitotic Pax6+ tRG cells were Olig2 negative and 18% expressed Olig2. By P10, the end of ferret cortical neurogenesis [19], the proportion of mitotic Pax6+ tRG cells that were Olig2 negative was 31% and the proportion that were Olig2+ increased to 62% (Figs. 18b & e).

In developing rat cortex mitotic Pax6+ tRG cells did not express Olig2 at E14. At E17 78% of mitotic Pax6+ tRG cells were Olig2 negative and 22% expressed Olig2. At P3, after cortical neurogenesis [21], the proportion of mitotic Pax6+ tRG cells that were Olig2 negative was only 3% and the proportion that were Olig2+ increased to 77% (Figs. 18c & f). We did not observe morphological differences between Pax6+ tRG cells that expressed Olig2 versus those that did not express Olig2.

We tested if the Olig2+ cells might be neurogenic by performing an Olig2/Tbr2 double immunostaining in the E21 rat but did not find any double positive cells (194 Tbr2+ cells, 432 Olig2+ cells), which suggests that Olig2+ precursor cells are restricted to producing glial progeny.

In these experiments testing for Olig2 expression by tRG cells relied on 4A4 identification of tRG cells. To test the validity of this approach we labeled tRG cells through in utero retroviral injections and electroporations of GFP-expressing plasmids. tRG cells were identified based on morphology, i.e.: cells in the SVZ that lacked a ventricular contacting process but that possessed an intact pial contacting process. We tested whether the tRG cells expressed Olig2 through immunostaining and found similar proportions of Olig2+ and Olig2-negative cells as we found among 4A4 identified tRG cells (data not shown).

These data are consistent the idea that two subtypes of tRG cells exist in the developing cortex during cortical neurogenesis, one subtype that expresses Olig2 and produces glial progeny, and a second subtype that does not express Olig2 and produces neuronal progeny. Furthermore, these data provide more evidence that the same cellular subtypes are present in the outer SVZ of lissencephalic rats that are present in the oSVZ of gyrencephalic ferrets and macaques.

### Identifying the oSVZ

**Figure 19:**
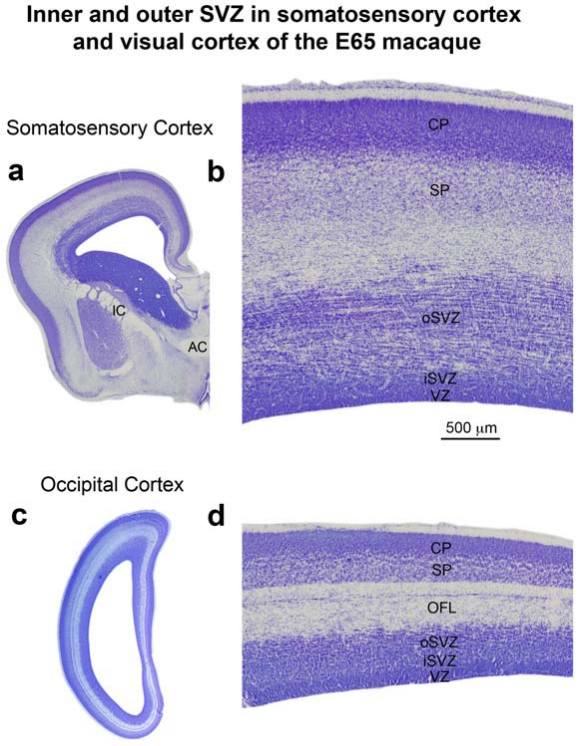
The outer fiber layer (OFL) is not present in cortical areas rostral to the occipital lobe. (**a, c**) Nissl-stained coronal sections of somatosensory and visual cortex from the E65 macaque. (**b,d**) Higher magnification images taken from the sections shown in (**a**) and (**c**). At E65 the OFL is visible in visual cortex superficial to the outer subventricular zone (oSVZ), but the inner fiber layer has not yet developed. The OFL is not apparent in somatosensory cortex. The oSVZ in somatosensory cortex is characterized by a striated appearance created by tangential streams of cells. Scale bar in (**b**) applies to (**d**). IC, internal capsule; AC, anterior commissure; VZ, ventricular zone; iSVZ, inner subventricular zone; SP, subplate, CP, cortical plate.

**Figure 20:**
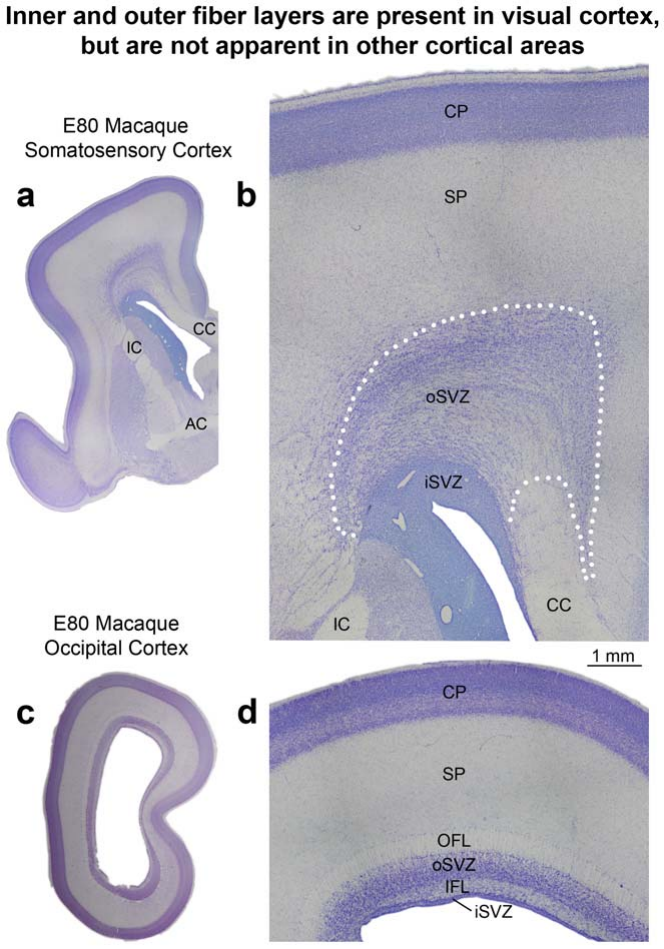
The inner fiber layer (IFL) and outer fiber layer (OFL) are present in the E80 macaque visual cortex but are not apparent in other cortical areas. (**a,c**) Nissl-stained coronal sections of somatosensory and visual cortex taken from E80 macaque. (**b,d**) Higher magnification images taken from the sections shown in (**a**) and (**c**). Dotted line in (**b**) represents the outer boundary of the outer subventricular zone (oSVZ) in somatosensory cortex determined through Tbr2 immunostaining on adjacent sections (see Fig. 5). The outer fiber layer (OFL) and inner fiber layer (IFL) are visible in the visual cortex but are not apparent in cortical areas rostral to the occipital lobe. In somatosensory cortex the boundary between the inner SVZ (iSVZ) and oSVZ can be visualized in Nissl stained tissue as a sharp border created by differences in cell density. In the visual cortex the IFL divides the iSVZ from the oSVZ. In somatosensory cortex the oSVZ extends much farther from the ventricle than it does in visual cortex and is characterized by a striated appearance. In the E80 macaque the ventricular zone has become very thin and the cell dense proliferative zone that surrounds the lateral ventricle consists almost entirely of iSVZ. The subplate (SP) was identified according to Smart et al 2002 [9]. Scale bar in (**b**) applies to (**d**). CC, corpus callosum; IC, internal capsule; AC, anterior commissure; CP, cortical plate.

The iSVZ and oSVZ were identified and subdivided into discrete proliferative zones in the macaque visual cortex based on cytoarchitectural features, particularly the inner and outer fiber layers [9]. The inner fiber layer becomes apparent in macaque visual cortex by E72 [9]. We compared cortical cytoarchitecture in Nissl stained coronal sections at three rostro-caudal levels and at several developmental stages in each species. We examined coronal sections of tissue at the genu of the corpus callosum, at the level of the anterior commissure, which includes primary somatosensory cortex in the ferret [19] and macaque [31], and in the occipital lobe. In Nissl stained sections of macaque tissue the inner and outer fiber layers were present in the occipital lobe (Fig. 4), as previously described [9]. However, these structures did not have the same appearance or were not apparent in somatosensory cortex or frontal cortical areas, and did not present obvious cues for distinguishing the iSVZ from the oSVZ (Figs. 19 and 20). At E65 a slight decrease in cell density was observed between the iSVZ and oSVZ in somatosensory cortex, but this was not apparent at E80. We also noted that the macaque oSVZ was much thicker in somatosensory cortex compared to visual cortex. One explanation for the difference in thickness may be that the somatosensory oSVZ generates cortical cells that are destined for wide spread cortical areas, including neocortex directly superficial to the somatosensory oSVZ and neocortical areas in the lateral cerebral wall (see Fig. 20a). In contrast, in the visual cortex there is a more strict “one-to-one’ radial relationship so that the oSVZ appears to serve only the developing cortex that is directly superficial (see Fig. 20c).

**Figure 21:**
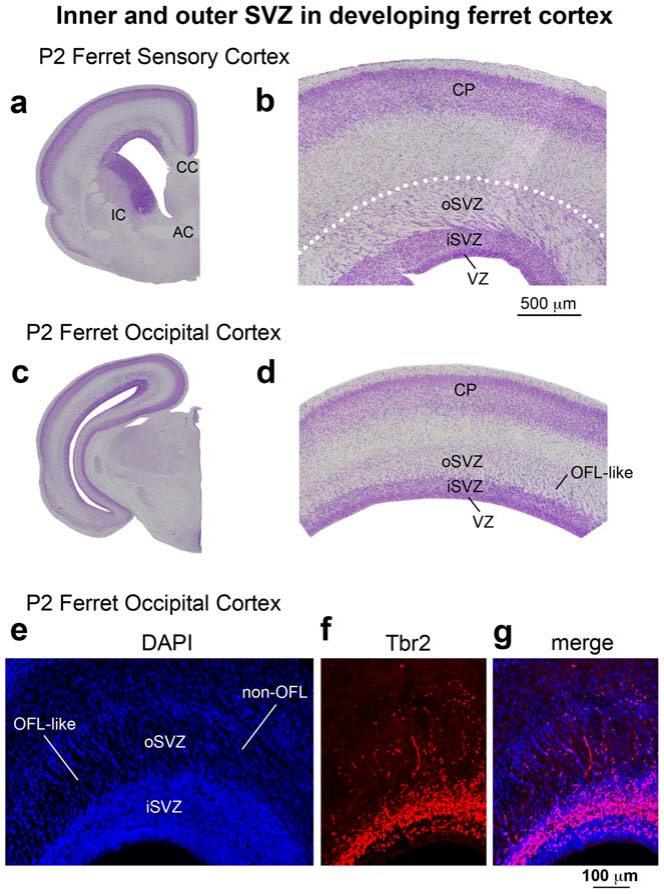
Inner and outer SVZ in the developing ferret cortex. (**a,c**) Nissl-stained coronal sections of somatosensory and visual cortex from P2 ferret. (**b,d**) Higher magnification images taken from the sections shown in (**a**) and (**c**). Dotted line in (**b**) represents the outer boundary of the outer subventricular zone (oSVZ) in somatosensory cortex determined through Tbr2 immunostaining. The outer fiber layer (OFL) and inner fiber layer are not present in somatosensory cortex, but a structure that resembles the OFL (OFL-like) is apparent in some areas of the ferret occipital lobe. However, note that the OFL-like structure in ferret is located within the oSVZ, in contrast to the OFL in macaque, which is superficial to the oSVZ. In both somatosensory and visual cortex the boundary between the inner SVZ (iSVZ) and oSVZ can be visualized in Nissl stained tissue as a sharp border created by different cellular density. The superficial boundary of the oSVZ can also be visualized in Nissl stained tissue based on cell density (determined through Tbr2 immunostaining). In somatosensory cortex the oSVZ is characterized by a striated appearance of cells organized into clusters that appear to stream at an oblique or tangential angle through the oSVZ. (**e–g**) The OFL-like structure in ferret resembles the macaque OFL since radially oriented clusters of cells stream through this structure as they do in macaque (see Fig. 4). But the ferret OFL-like structure differs from the macaque OFL since it is within the oSVZ rather than superficial to the oSVZ and because Tbr2+ cells are located throughout the OFL-like structure in ferret but do not penetrate the macaque OFL (See Fig. 4). VZ, ventricular zone; CP, cortical plate. Scale bar in (**b**) applied to (**d**). Scale bar in (**g**) applied to (**e**, **f**).

**Figure 22:**
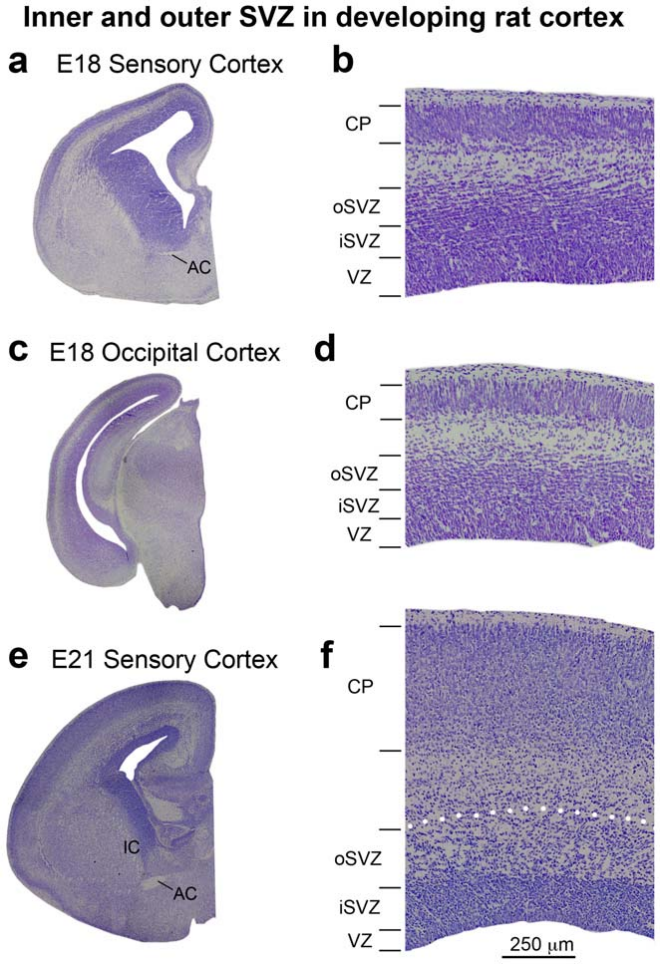
Inner and outer SVZ in the developing rat cortex. (**a, c, e**) Nissl-stained sections of somatosensory or visual cortex from the E18 rat. (**b, d, e**) Higher magnification images taken from the sections shown in (**a**), (**c**) and (**e**). The inner fiber layer and outer fiber layer are not apparent in any cortical areas of rat cortex. At late stages of rat cortical development, such as E18 or E21, the boundary between the inner subventricular zone (iSVZ) and outer SVZ (oSVZ) can be discriminated based on differences in cell density. At E17 and younger ages Tbr2 immunostaining is required to visualize the location of dense inner band of Tbr2+ cells to localize the iSVZ and oSVZ. The oSVZ is characterized by a stippled or striated appearance created by oblique and tangential clusters of cells that appear to stream across the oSVZ. At E21, the oSVZ has expanded compared to earlier stages of cortical development. Dotted line indicates the upper boundary of the oSVZ. AC, anterior commissure; IC, internal capsule; VZ, ventricular zone; CP, cortical plate. Scale bar below (**f**) applies to (**b, d, f**).

We noted that the inner fiber layer was not apparent at any age in somatosensory cortex in Nissl stained tissue prepared from ferret. In some areas of the ferret visual cortex, cells in the oSVZ were organized in radial palisades that resembled the organization of the macaque OFL (Fig. 21, see Fig. 4). However, this ‘OFL-like’ structure in ferret differed from the macaque OFL since it is superficial to the iSVZ, not the oSVZ, and because Tbr2+ cells penetrate through the ferret ‘OFL-like’ structure, whereas Tbr2+ cells are not present in the macaque OFL (Figs. 21 and Fig. 4). The radially oriented streams of cells in the OFL-like structure of ferret visual cortex likely represent cell migration along a radial trajectory. The oSVZ in many cortical areas of ferret and rat was characterized by streams of cells that appear to cross the oSVZ along oblique or tangential trajectories (Figs. 19, 20, 21, 22). The organization of these cells is likely influenced by a combination of factors including the migratory trajectory of newly generated cells as well as the ingrowth of axonal fibers. Nevertheless, the IFL and OFL were not apparent in any cortical areas of the developing rat cortex (Fig. 22).

**Figure 23:**
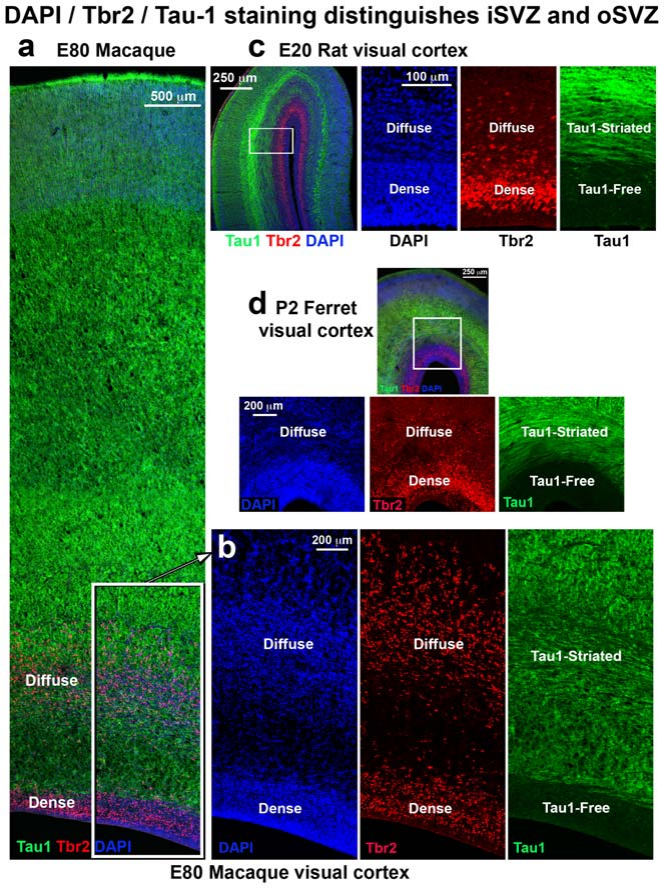
Tau-1 and Tbr2 staining distinguishes the inner subventricular zone (iSVZ) from the outer SVZ (oSVZ). (**a**) A coronal section from E80 macaque somatosensory cortex immunostained for Tau-1 (green), Tbr2 (red), and counterstained with DAPI (blue). Tau-1 staining produces dense labeling in the cortical plate, striated staining in the outer subventricular zone (oSVZ) and an absence of label in the inner SVZ (iSVZ). The striated appearance of Tau-1 staining in the oSVZ complements the striated pattern of the oSVZ that is seen in Nissl or DAPI stained tissue. (**b**) Inset from (**a**) showing the pattern of cell density (DAPI, blue), Tbr2 staining (red), and Tau-1 staining (green). The Tau-free zone corresponds to the cell dense iSVZ/VZ visualized in DAPI staining (blue) and where the dense inner band of Tbr2+ cells (red) is located. The boundary between the Tau-free and the Tau-striated zone delineates the boundary between the iSVZ and oSVZ where the diffuse outer band of Tbr2+ cells is located. (**c**) Tau-1 and Tbr2 immunostaining produces the same pattern in the E20 rat. The Tau-free zone corresponds to the cell dense VZ/iSVZ visualized in DAPI staining (blue) and where the dense inner band of Tbr2+ cells (red) is located. The boundary between the Tau-free and the Tau-striated zone delineates the boundary between the iSVZ and oSVZ where diffuse Tbr2+ cells are located. (**d**) Tau-1 and Tbr2 immunostaining produces the same pattern in the P2 ferret. The Tau-free zone corresponds to the cell dense VZ/iSVZ visualized in DAPI staining (blue) and where the dense inner band of Tbr2+ cells (red) is located. The boundary between the Tau-free and the Tau-striated zone delineates the boundary between the iSVZ and oSVZ where diffuse Tbr2+ cells are located.

**Figure 24:**
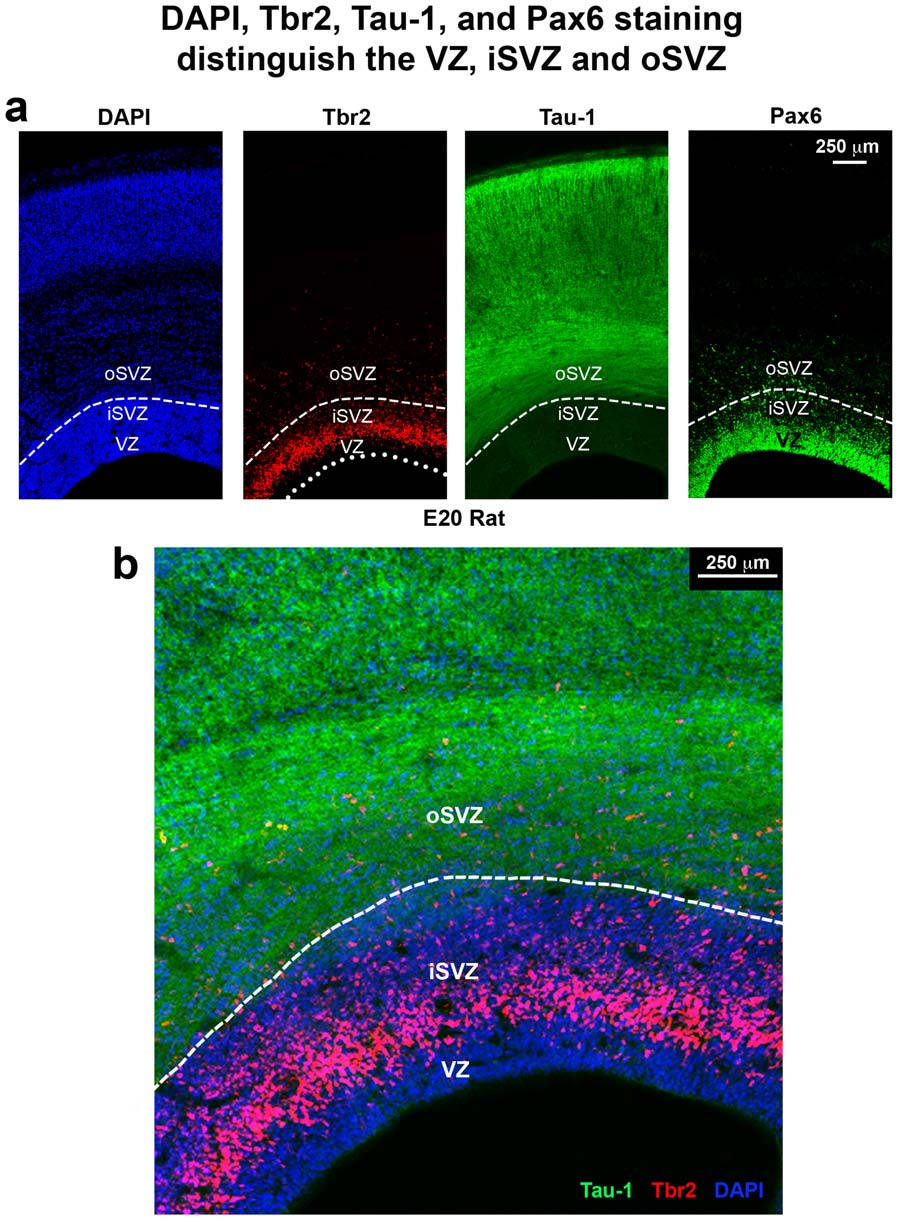
The boundaries between the ventricular zone (VZ), inner subventricular zone (iSVZ) and outer SVZ (oSVZ) can be identified through a combination of DAPI, Tau-1, Tbr2 and Pax6 staining. (**a**) Coronal sections of somatosensory cortex from E20 rat stained with (from left to right) DAPI (blue), and immunostained for Tbr2 (red) and Tau-1 (green). The right panel is an adjacent section that was immunostained for Pax6 (green). DAPI staining identifies the boundary between the iSVZ and oSVZ. The VZ and iSVZ have a near uniform cell density, while the oSVZ is striated and the cell density is lower. Tau-1 staining also identifies the boundary between the iSVZ and oSVZ in macaque, ferret and rat. The iSVZ is located in the Tau-free zone. The border between iSVZ and oSVZ is delineated by the border between the Tau-free zone and the Tau-striated zone. Tau-1+ fibers are largely absent from the VZ and iSVZ. Tbr2 and Pax6 can be used to discriminate the VZ and iSVZ. The dense inner band of Tbr2 expression corresponds to the iSVZ and the dense inner band of Pax6 expression corresponds to the VZ. The dashed lines indicate the boundary between the iSVZ and oSVZ.

We asked if the inner and outer fiber layers could be better visualized in other cortical regions of macaque, ferret, and rat neocortex through immunohistochemistry using Tau-1 antibodies that label axonal fibers. Tau-1 immunostaining in the macaque produced dense immunoreactivity in the cortical plate and developing white matter, a striated pattern of staining in the oSVZ, and scarce labeling in the iSVZ and VZ (Fig. 23a & b). The same pattern was present in rats and ferrets (Fig. 23c & d). Tau-1 immunostaining did not provide evidence for an inner or outer fiber layer in frontal/somatosensory sections of macaque cortex, or in any regions of ferret and rat cortex. However, in sections that were co-stained with Tau-1 and Tbr2 antibodies we noticed striking similarities in the pattern of staining in rat, ferret and macaque. In each species the dense inner band of Tbr2 expression was located in the Tau-free zone. In macaque, ferret, and rat the diffuse band of Tbr2 expression was located in the Tau-striated zone. In some regions of macaque cortex the diffuse Tbr2+ cells extended beyond the horizontally oriented fibers in the Tau-striated zone. The Tau-free zone corresponded precisely with the cell dense proliferative region that surrounds the ventricle in rats, ferrets, and macaques, and the Tau-striated zone corresponded to the more superficial zone that has a lower cell density, as seen in DAPI, Nissl or other cellular stains (Fig. 23). The changes in cell density between the iSVZ and oSVZ, the absence of Tau-1+ fibers in the iSVZ, and the presence of Tau-1+ fibers in the oSVZ of each species demonstrate that the iSVZ and oSVZ subdivisions are histologically distinct structures in macaque, ferret, and rat. Thus, Tau-1 immunostaining can be used in conjunction with DAPI, and Tbr2/Pax6 to distinguish the boundaries between the VZ, iSVZ and oSVZ proliferative zones in these three species (Figs. 23 and 24). It is important to note that although Tbr2 staining in the E17 rat shows the presence of a dense inner band and diffuse outer band, the distinction between iSVZ and oSVZ based on cell density alone is not discernible until E18, when perceptible changes in cell density between the iSVZ and oSVZ become readily apparent.

In summary, Pax6 immunostaining presented as a dense band that corresponds to the VZ, and a diffuse band of labeled cells in the iSVZ and oSVZ, with a few scattered cells in the CP and MZ. Tbr2 immunostaining presented as a dense band that corresponds to the iSVZ and a diffuse band of labeled cells in the oSVZ. DAPI staining identified the cell dense proliferative zone around the ventricle that includes the VZ and the iSVZ, and a lower density proliferative zone that includes the oSVZ. Finally, Tau-1 immunostaining distinguished the border between iSVZ from the oSVZ since Tau-1+ fibers do not enter the cell dense proliferative region of the VZ/iSVZ (Fig. 24). In macaque the dense band of Tbr2+ cells nearly fills the Tau-free/cell dense proliferative region. However, in ferret and rat the inner dense band of Tbr2+ cells does not completely fill the iSVZ, and the diffuse band of Tbr2+ cells is located in the outer portion of the iSVZ and in the oSVZ. We define the rat oSVZ as the proliferative region where Tbr2+ cells are localized superficial to the cell dense iSVZ (Fig. 24a). We propose that in later stages of rat cortical development, the SVZ is divided into an iSVZ and oSVZ, as it is in macaque and ferret. The boundary between iSVZ and oSVZ can be identified based on cell density in Nissl or DAPI stained tissue, and/or Tau-1 immunostaining. These data support the hypothesis that the developing rat cerebral cortex possesses distinct iSVZ and oSVZ compartments.

## Discussion

We provide evidence that the lissencephalic rat cortex possesses an outer SVZ. We show that the embryonic SVZ in rat, ferret and macaque shares key cellular and cytoarchitectural features. We demonstrate a simple novel approach for discriminating the boundaries of the VZ, iSVZ and oSVZ in mammals based on costaining with DAPI, Tbr2/Pax6, and Tau-1. We also describe the presence of two subtypes of tRG cells that co-exist in the perinatal neocortex based on the expression of Olig2.

### Defining the outer SVZ: does size matter?

The cerebral cortex of macaques is much more extensive than that of rats or ferrets. This difference is reflected in total brain weight. The adult rat brain weighs approximately 2 grams, the adult ferret brain weighs approximately 7 grams and the adult macaque brain weighs 90–100 grams [32]. Furthermore, there are approximately 100 times more neurons in the macaque neocortex than in the rat (approximately two billion neurons in macaque [33], [34], versus 20–30 million neurons in rat [35]–[37]). The number of neurons in the ferret neocortex has not been determined, but based on brain weight one might predict that ferret numbers lie much closer to rat than to macaque values. The oSVZ in macaque is considerably larger than the oSVZ in ferret or rat, up to 20 times thicker than the rat oSVZ in developing somatosensory cortex. In addition, the macaque oSVZ is populated by a significantly greater number of precursor cells than the oSVZ of ferret or rat (Figs. 7 and 10). Thus, based on a number of measures the macaque oSVZ and resulting neocortex are significantly larger than in rats or ferrets. However, the determination of whether or not the oSVZ is present in a given species should be based on a defined set of characteristics rather than sheer size or number of cells.

We propose a definition for the iSVZ and oSVZ based on the following criteria: 1) The iSVZ is located within the cell dense proliferative region that surrounds the lateral ventricle, while the oSVZ is located in the cell-diffuse zone that is superficial to the iSVZ; 2) The iSVZ appears homogeneous in terms of cell density, while the oSVZ is often marked by a striated or stippled appearance created by clusters of cells organized into oblique or tangential streams that appear to cross the oSVZ; 3) The iSVZ is localized to the dense inner band of Tbr2+ cells and the oSVZ is localized to the diffuse outer band of Tbr2+ cells; 4) The iSVZ is localized to the Tau-free zone that surrounds the ventricle, the boundary between the iSVZ and oSVZ is marked by the boundary between the Tau-free and Tau-striated zones, and the oSVZ is localized in the Tau-striated zone; 5) The iSVZ and oSVZ are marked by the presence of mitotic Pax6+ tRG cells; and 6) The iSVZ and oSVZ are sites of neurogenesis. We have shown that the iSVZ and oSVZ of macaque, ferret and rat meet these cytoarchitectural criteria.

Neurogenesis in the macaque and ferret oSVZ has not been directly visualized but is widely accepted based on the presence of Tbr2+ cells in the oSVZ. We show here that Tbr2+ mitoses, the majority of which are presumed to be neurogenic divisions [4], [16], [38], are located throughout the macaque and ferret iSVZ and oSVZ (Fig. 11). Evidence for neurogenesis in the rodent iSVZ has been conclusively demonstrated by multiple laboratories over the past decade [4], [5], [16], [38]–[40]. Evidence for neurogenesis in the structure we term oSVZ in the rat has been reported. We previously showed through time-lapse imaging of in vitro slices prepared from embryonic rats that intermediate progenitor cells migrate up to 175 µm from the ventricle before undergoing divisions that produce neurons that we identified through electrophysiological recordings [5]. Miyata and colleagues have shown cell divisions occurring at similar distances from the ventricle in developing mouse cortex [16]. In the current study we show in vivo that Tbr2+ mitoses are located up to 300 µm from the ventricle. These mitotic cells are superficial to the cell dense proliferative zone that surrounds the lateral ventricle and are located within the structure we term the oSVZ (see Fig. 22). We conclude that the rodent cortex is subdivided into an iSVZ and an oSVZ during later stages of cortical neurogenesis. In rat the oSVZ can be distinguished from the iSVZ starting at E18, which is the peak of neurogenesis for layer 3 neurons in the rat somatosensory cortex [21]. An important point that must be addressed concerns the nature of the iSVZ versus the oSVZ. Are these structures functionally distinct? Histologically the iSVZ and oSVZ are distinct (see Figs. 20, 21, 22, 23, 24). In addition, the proportion of mitotic cells in each compartment that express markers such as Tbr2 differs in the iSVZ and oSVZ (see Table 9), demonstrating that each zone comprises a different cohort of precursor cells. However, whether or not neural progenitor cells in the iSVZ and oSVZ are functionally different remains to be determined.

### Does the outer SVZ produce gyrencephalic cortex?

We examined the expression characteristics and distribution of precursor cells in the developing somatosensory cortex of mammals representing 3 orders: rodents (rat), carnivores (ferret), and primates (macaque). We expected to find more commonalities in the developmental organization of the SVZ in the gyrencephalic ferret and macaque, but instead found that lissencephalic rat and gyrencephalic ferret shared more in common. Our analyses showed that the distribution of Tbr2+ cells and Pax6+ cells, and mitotic cells in general, shift away from the ventricle to the oSVZ during early stages of macaque cortical neurogenesis. In contrast, the shift of precursor cells away from the ventricle to the oSVZ occurred much later in rats and ferrets and was not as pronounced. For example, in macaque the majority of Pax6+ cells shift to the oSVZ prior to genesis of layer 5 neurons, but in rats and ferrets the majority of Pax6+ cells remain in the VZ throughout cortical neurogenesis (Fig. 9). These results suggest that the shift of precursor cells to the oSVZ that occurs in macaque may not be a prerequisite for the development of gyrencephalic cortex since it does not occur in ferrets.

The size of oSVZ is unquestionably a key determinant for the large number of cortical neurons in primate neocortex. In fact, we find that the macaque oSVZ is much thicker and has considerably more Tbr2+ cells than the ferret or rat oSVZ (see Fig. 8). However, it does not necessarily follow that the oSVZ is a crucial factor in the development of gyrencephaly, especially when considered in light of the differences we found between characteristics of oSVZ in ferret and macaque. Regional differences in the thickness of the SVZ have been proposed as one mechanism that could play a factor in gyrencephaly [41]. This hypothesis, which remains to be thoroughly tested, posits that gyri form over regions of the SVZ that are thicker while sulci form over regions of the SVZ that are thinner. Alternatively, changes in the level of cell production in the proliferative zones could be associated with gyrus and sulcus formation. In support of this idea, Reillo and colleagues (2011) reported that proliferation in the oSVZ is two to four times greater in regions of the developing ferret neocortex that produce gyri versus regions underlying sulci [42]. Mechanisms that could harness and regulate the proliferative output of precursor cells in the VZ and/or SVZ on a regional basis have been proposed (e.g.: [43]), but remain to be fully understood. Other mechanisms may work in parallel to shape the formation of gyri and sulci. For example, Van Essen's tension-based hypothesis posits that axonal connections between cortical areas define the patterns of sulci and gyri [44]. We believe that cell proliferation plays an important role in the formation of gyri but cannot rule out the possibility that multiple mechanisms work simultaneously in the developing forebrain to produce the complex pattern of sulci and gyri that characterize gyrencephalic neocortex.

### Gliogenic and neurogenic tRG cells are present in the developing rat neocortex

We found that Pax6-expressing tRG cells are present in the developing rat neocortex as in ferret and macaque (Figs. 14, 15, 16). We have previously shown that translocating radial glial cells are present in the embryonic rat, that individual tRG cells in the embryonic brain divide multiple times and produce daughter cells that lack neuronal membrane properties [4], [5]. Other groups have also investigated mitotic tRG cells and reported that they produce neuronal progeny based on marker expression [16], [17]. We show that tRG cells express Pax6 and Sox2 in the rats and mice as they do in ferret and macaque (Fig. 17), and as has been reported in the mouse [17], [18]. We further these findings by showing that mitotic Pax6-expressing tRG cells are not restricted in function to neurogenesis since they are present in the neocortex after cortical neurogenesis is complete (Fig. 16). We also show that tRG cells comprise multiple subtypes based on the expression of Olig2, which has been shown to direct astrocyte formation in the postnatal SVZ [30]. We show that during neurogenesis in the macaque, ferret and rat the majority of mitotic tRG cells are Pax6+ and Olig2-negative, but that by the end of neurogenesis the majority of mitotic tRG cells are Pax6+/Olig2+ (Fig. 18). We propose that these subtypes represent neurogenic and gliogenic precursor cells, respectively.

### tRG cells are a common cell type in mammalian neocortex

RG cells have long been known to translocate away from the ventricle and transform into astrocytes [12]–[15]. Recent work has shown translocating RG cells are present in humans [10], [11], non-human primates including lissencephalic primates [45], carnivores such as ferrets [11], [46], and rodents [17], [18]. Furthermore, recent work shows that translocating radial glial cells in the oSVZ divide and produce neuronal and glial daughter cells. Multiple names have been applied to these cells including outer RG (oRG) cells [10], oSVZ progenitor cells [11], intermediate radial glia [42], outer VZ progenitor cells [18], and basal radial glia [47], [48]. In all cases the translocating cells share a core set of characteristics: maintenance of the pial process, translocation away from the ventricle toward the overlying cortical plate, loss of the ventricular contacting process, and asymmetric cell division that produces self renewed Pax6+ translocating radial glial cells. We suggest that these cells, meticulously described by different laboratories in several mammalian species, belong to a single cell class and we propose the simplified term ‘tRG’ to encompass members of this cell class. This term reflects the key characteristics that identify tRG cells - translocation and radial glial identity. Furthermore, ‘tRG’ reflects the fact that these cells translocate through and are present in the VZ, the iSVZ, and the oSVZ. It is likely that subtypes of tRG cells exist within this class. Indeed, recent studies have advanced our understanding of the functions of tRG cells [10], [15], and have also shown that the tRG cell class includes diverse subtypes that can be identified based on marker expression [44]. In addition, our current results demonstrate that the transcription factor Olig2 can be used to distinguish two Pax6+ tRG cell subtypes (Fig. 19): those that presumably generate neurons and those that presumably generate astrocytes. Future studies should more closely examine the commonalities and differences between the various translocating cells that have been identified in the mammalian neocortex.

Some recent studies have proposed that tRG cells are unique to the developing primate cortex, and are sparsely present in rodents. However, our analysis shows that tRG cells are also quite prominent in rodents. Our current results match previous studies in which we (Noctor et al., 2004, 2008) and others (Miyata et al., 2004) demonstrated through detailed time lapse analysis the presence of mitotic translocating RG cells in the embryonic rodent neocortex that maintain their pial process, translocate away from the ventricle and undergo asymmetric divisions that yield self-renewed RG cells that retain translocating morphology [4], [5], [16]. We invite interested parties to view detailed time-lapse movies of translocating RG cells in the embryonic rat in online supplemental information accompanying our previous publications [4], [5]. In addition, we present new data showing that Pax6+ tRG cells are still present in the postnatal rat neocortex at P3, and are present after neurogenesis has ended in P10 ferret and E151 macaque somatosensory cortex (Fig. 16). The function of mitotic tRG cells in the post-neurogenic neocortex is presumably related to gliogenesis, but this remains to be confirmed.

### Identifying the inner and outer SVZ

Seminal work that first described the oSVZ was performed in the macaque visual cortex [9]. In the macaque visual cortex the inner fiber layer (IFL) divides the iSVZ and oSVZ by age E78, and the outer fiber layer (OFL) serves as the superficial boundary for the oSVZ [9]. We found that the IFL and OFL were not apparent in coronal sections of the somatosensory cortex or other cortical areas in the frontal lobe (Figs. 5, 19 and 20). Smart et al., (2002) reported that the IFL extends from the ganglionic eminence to the pole of the occipital lobe [9]. At E65 in macaque somatosensory cortex we observed a slight decrease in cell density between the iSVZ and oSVZ (Fig. 19), but the IFL was not present at E80 in somatosensory cortex (Fig. 20). The IFL and OFL were not apparent at any age in ferret or rat (Figs. 21 and 22). We developed a new approach for identifying the boundaries between the VZ, iSVZ and oSVZ in any cortical region of the rat, ferret and macaque based on DAPI, Tbr2/Pax6, and Tau-1 staining (Fig. 24). This method makes identification of the iSVZ and oSVZ an unambiguous task.

### Conclusion

Rodents and primates are both in the superorder Euarchontoglires while ferrets are in the superorder Laurasiatheria. Therefore macaques are more closely related to lissencephalic rats than they are to gyrencephalic ferrets. This suggests that gyrencephalic cortex may have evolved independently in ferrets and macaques, or alternatively that a blueprint for cortical expansion was present in ancestral mammalian species. We prefer the second hypothesis and propose that this ‘blueprint’ is the oSVZ. We show that rats possess an oSVZ, albeit limited in size and only present during late stages of cortical neurogenesis. Understanding how expansion of this structure occurred, for example through integrin signaling [11], and how its neurogenic function is maintained over a longer developmental period in some species will shed light on mechanisms that drove evolution of the human cerebral cortex. The complexity of laminar structures in the developing rat neocortex has been described in detail by Bayer and Altman [21], and the functional significance of the rat proliferative zones has become clear during the past decade as precursor cell types have been identified. Perhaps one of the greatest differences between rodent and primate cortex is one of quantity, not quality. We conclude that the developing rodent cerebral cortex shares key features with that of primates, and therefore serves as a valid model for understanding human cortical development under normal and pathological conditions.

## Materials and Methods

### Animals

Procedures on rodents (Sprague Dawley rats and Swiss Webster mice) were approved by the Institutional Animal Care and Use Committee of the University of California, Davis and were conducted in accordance with the National Institutes of Health guidelines for the use of animals in research (Protocol #16368). Ferret tissue (*Mustela putorius*) was collected by the laboratories of Drs. Francisco Clasca, Sharon Juliano and Barbara Chapman for other purposes and sections of brain tissue were provided for this study as a generous gift. Macaque tissue (*Macaca mulatta*) was collected by the laboratory of Dr. David Amaral for another purpose [49], and sections of brain tissue were provided for this study as a generous gift. Procedures on macaques were approved by the Institutional Animal Care and Use Committee of the University of California, Davis (protocol # 12139), and strictly adhered to National Institutes of Health policies on primate animal subjects. Rat brains that were used included embryonic day (E)13, E14, E17, E18, E20, E21 and postnatal day (P)1, P2, P3, P7, and P10. E18 mice brains were used. Ferret tissue was obtained from animals at E23, E28, E31, E34, E38, P2, and P10. Macaque tissue was obtained from animals at E50, E65, E80, E100 and E151. For rats, ferrets and macaques these ages encompassed the neurogenic and early gliogenic phase for somatosensory cortex in each species [19]–[21].

### Tissue Processing

All animals were transcardially perfused with 4% paraformaldehyde (PFA) with the exception of E14 rats and E23 ferrets, which were immersed in 4% PFA overnight. Brains were removed, post-fixed in 4% PFA for two hours, cryoprotected overnight in 30% sucrose, sectioned coronally on a cryostat, mounted on Superfrost Plus slides (Fisher) and stored at −20°C until use.

### Preparation of Retrovirus

Replication-incompetent pantropic retrovirus encoding enhanced green fluorescent protein (eGFP) was prepared as described previously [3]. 293gp NIT-GFP cells were grown to 70–80% confluency and transiently transfected with pVSV-G (Clontech, generous gift of Dr. James Angelastro) using Lipofectamine 2000 (Invitrogen) or CalPhos Mammalian Transfection Kit (Clontech) to pseudotype viral particles. Supernatant was collected at 48 hrs post transfection and virus was concentrated by centrifugation at 50,000×g for 1.5 hours at 4°C, resuspended in Opti-MEM (Invitrogen), and stored at −80°C until use.

### Nissl Staining

Cryostat-sectioned coronal sections (14 µm) from embryonic rats, ferrets and macaques were mounted on Superfrost Plus slides. Slides were hydrated in a series of two minute incubations as follows: 100% ethyl alcohol (EtOH), 96% EtOH, 70% EtOH, 50% EtOH, and two incubations in Milli-Q H_2_O. Slides were then incubated for two minutes in a 0.1% cresyl violet solution. Slides were dehydrated in a series of two minute incubations as follows: Milli-Q H_2_O, Milli-Q H_2_O, 50% EtOH, 70% EtOH, and 96% EtOH. Slides were then incubated in 100% chloroform for two minutes on a shaker. Cresyl Violet stain was differentiated in 95% EtOH+glacial acetic acid until nucleoli were clearly visible (2–10 minutes). Slides were placed in 100% EtOH for two minutes, incubated in Safeclear (Fisher) for five minutes and coverslipped in DPX mounting medium (EMS).

### In Utero Intracerebral Retroviral Infection

Pregnant rats at gestation days E16 through E20 were anesthetized with 3–5% Isoflurane, a laparotomy was performed, and the uterine horns containing embryos were temporarily removed. A solution containing concentrated retrovirus was injected into the lateral ventricles of embryos and uterine horns were replaced in the peritoneal cavity. During injections the uterine horns were lavaged with warm artificial cerebral spinal fluid (aCSF) containing (in mM): NaCl 125, KCl 5, NaH_2_PO_4_ 1.25, MgSO_4_ 1, CaCl_2_ 2, NaHCO_3_ 25, and glucose 20, pH 7. The peritoneal cavity was lavaged with aCSF and the incision was sutured. The mother was allowed to recover from anesthesia and treated with Buprenorphine for analgesia. Embryos were allowed to develop for 1–16 days (E17 - P10), removed from the mother and some embryos were transcardially perfused with 4% PFA. Prior to perfusion postnatal animals were anesthetized using ice-cold anesthesia according to UC Davis IACUC guidelines. Brains were removed, cryoprotected in 30% sucrose, and sectioned on a cryostat. Cells with morphology of tRG cells were identified, and slices containing cells were processed for immunohistochemistry (see below).

### Time-Lapse Recording of tRG Cells

One to four days post injection the pregnant mother was anesthetized with Isoflurane, a laparotomy was performed and uterine horns exposed. Embryos were removed one at a time and decapitated on ice. Brains were removed, embedded in 4% Low Melting Point Agarose (Promega) and sectioned at 400 µm on a vibratome (Ted Pella) in bubbling carbogenated (95% O_2_/5% CO_2_) aCSF. Slices were placed in bubbling carbogenated aCSF until all brains were sectioned. Sections were then transferred to six-well plates containing Millicell culture inserts (Millipore) and slice culture media containing (v/v): 66% BME, 25% HBSS, 5% Fetal Bovine Serum, 1% N-2 Supplement, 1% Pen/Strep/Glutamine (Invitrogen) and (w/v) 0.66% D+ Glucose (Sigma). Slices were placed in a humidified, 37°C incubator containing ambient oxygen and 5% CO_2_ and allowed to equilibrate for four hours. Six-well plates containing slices were removed from the incubator and cells were imaged on an Olympus FV1000 confocal microscope at 2–3 hour intervals for up to 84 hours. Projection images were made from Z-stacks that included all visible processes of individual GFP+ cells, and all GFP+ cells in individual clones on a PC running Fluoview (Olympus). Laser power was monitored to prevent phototoxicity. Transmitted light images of the slices were taken periodically to track cell movements within the slices. Time-lapse sequences were reconstructed using Photoshop (Adobe). The position of labeled cells was registered at each timepoint using transmitted light images, ventricular surface, adjacent clones, and the pial surface. After time-lapse recording, slices were fixed in 4% PFA for 15 minutes at RT, cryoprotected in 30% sucrose, sectioned at 50 µm on a cryostat, mounted on slides and processed for immunohistochemistry (see below).

### In utero electroporation

Timed pregnant mice at E16 were anesthetized as above. A laparotomy was performed and the uterine horns were removed from the peritoneal cavity. In utero intracerebral injection of pEGFP-N1 (1.0 µg/µL, Clontech) was performed into the lateral ventricles of the embryos. Tweezertrode electrodes (Harvard Apparatus) were positioned with the positive electrode over the dorsal cortex of the embryos. A square wave electroporator (Harvard Apparatus) was used to drive five pulses at 50 ms/pulse, 30 volts/pulse, with a 1 second interval between pulses. After electroporation, the uterine horns were replaced and lavaged as above. The incision was sutured, and the mother was given Buprenorphine for analgesia. The mother was allowed to recover from anesthesia and survived until E18, when the embryos were removed, perfused and cryosectioned as above.

### Immunohistochemistry

Tissue was prepared and mounted on slides as above. Antigen retrieval was performed on slide-mounted tissue by boiling sections in 10 mM Citrate Buffer (pH 6.0) containing 10 mM Citric Acid (Fisher) and (v/v) 0.5% Tween-20 (Acros) for fifteen minutes. Sections were blocked in blocking buffer containing (v/v) 10% fetal donkey serum, 0.1% Triton X-100 (Acros), and (w/v) 0.2% gelatin (Acros) for a minimum of one hour at room temperature (RT). Sections were incubated in primary antibody buffer containing primary antibodies (see below), (v/v) 2% fetal donkey serum, 0.02% Triton X-100, and (w/v) 0.04% gelatin overnight at RT. Primary antibodies included mouse anti-phosphorylated vimentin (4A4) 1∶500 (MBL), mouse anti-Pax6 1∶1 (Developmental Studies Hybridoma Bank), mouse anti-Pax6 1∶50 (Abcam), mouse anti-Sox2 1∶50 (R&D Systems), mouse anti-Tau-1 1∶200 (Millipore), mouse anti-NeuN 1∶200 (Millipore), rabbit anti-Pax6 1∶100 (Covance), rabbit anti-Tbr2 1∶500 (Abcam), rabbit anti-Phosphohistone H3 (PH3) 1∶50 (Chemicon), goat anti-Olig2 1∶500 (R&D Systems), chicken anti-GFP 1∶500 (Abcam) and rat anti-BrdU-FITC 1∶50 (Abcam). Sections were rinsed in 0.1 M PBS, then incubated for one hour at RT in secondary antibody buffer, which contained secondary antibodies (see below), (v/v) 2% fetal donkey serum, 0.02% Triton X-100, (w/v) 0.04% gelatin, and DAPI 1∶1000 (Roche). Secondary antibodies were conjugated to Dylight 405, Cy2/Dylight 488, Cy3/Dylight 549, or Cy5/Dylight 649 (Jackson Laboratories) and included donkey anti-mouse, donkey anti-rabbit, donkey anti-chicken, donkey anti-goat and donkey anti-rat. Slices were then rinsed and coverslipped with Mowiol.

### Analysis of Number of Mitoses by Nissl staining, 4a4 Immunostaining and PH3 Immunostaining

Mitoses were identified in Nissl-stained tissue by identifying condensed chromatin. Alternatively, mitoses were identified in tissue immunostained for 4A4 or PH3 and confirmed by condensed chromatin with DAPI counterstaining. At least three adjacent sections were analyzed from three different locations in the rostrocaudal axis (nine sections from each brain). Rostral sections were anterior to the genu of the corpus callosum, middle sections were at the level of the anterior commissure and/or interventricular foramen, and caudal sections were located caudal to the hippocampal formation in the occipital lobe. Mitoses were quantified in the entire dorsal cortical wall from the dorsomedial boundary with cingulate cortex to the corticostriatal junction. Mitoses were allocated to discrete histological zones (ventricular zone (VZ), inner subventricular zone (iSVZ), outer subventricular zone (oSVZ), or cortical plate (CP)/preplate (PP)/subplate (SP)/marginal zone (MZ)) based on cytoarchitecture of the region.

### Analysis of Density of Tbr2+ Cells in the Tbr2+ Dense Inner Band and Diffuse Outer Band

The dense inner band and diffuse outer band of Tbr2-expressing cells were identified in combination with DAPI staining. All Tbr2+ cells in a 2500 µm^2^ region of interest (ROI) located in the dense inner band and the diffuse outer band were quantified. A minimum of nine separate ROIs from the Tbr2+ dense inner band and diffuse outer band were averaged at each age in each species. Analysis was performed in the somatosensory cortex of rats (E14, E17, E18, E20), ferret (E28, E31, E34, E38, P2) and macaque (E50, E65, E80, E100).

### Quantification of Total Pax6+ and Tbr2+ cells

Coronal sections of rat, ferret, and macaque tissue immunostained for Pax6 or Tbr2 were imaged on a confocal microscope (Olympus). A montage of a cortical radial unit was created in Photoshop (Adobe) by combining individual optical sections from a series of overlapping high magnification (40×) Z-stacks. A 200 µm wide bin was created that stretched from the ventricular surface to the pial surface in the dorsal somatosensory cortex. All Pax6+ or Tbr2+ cells in the bin were counted, and allocated into discrete histological zones (VZ, iSVZ, oSVZ or CP/PP/SP/MZ) that were identified by DAPI staining. The number of positive cells/bin and the relative distribution of Pax6+ and Tbr2+ cells were compared across ages and species.

### Quantification of Double Immunolabeling in Mitoses

Rat, ferret and macaque sections that were double-immunostained using a variety of antibodies and counterstained with DAPI were imaged on a confocal microscope (Olympus). Z-stacks were created from images constructed from optical sections in which penetration of all antibodies and DAPI was complete. All mitoses, identified by condensed chromatin (DAPI) and 4A4/PH3 immunostaining, were identified in Z-stacks and the extent of costaining with different antibodies was assessed. Mitoses were allocated to discrete histological zones using DAPI staining as described above. The proportions of mitoses that immunostained positive or negative with specific antibodies were quantified for each area. The distribution of cells expressing 4A4/Pax6 and 4A4/Tbr2 was analyzed by quantifying the proportion of 4A4+/Tbr2+ and 4A4+/Pax6+ cells in each histological zone in a 300 µm wide radial unit of cortex that stretched from the ventricle to the pial surface.

### BrdU Birthdating

Postnatal rats received two intraperitoneal injections of BrdU (80 mg/kg, Sigma) in the morning and evening on P1, P3 or P7 to label cells born on that day. Injected pups survived until P10. Animals were perfused transcardially with PFA, cryoprotected and cryostat sectioned as described above. Cryostat sections were washed in 0.1 M PBS, incubated in 2 N HCL for 30 minutes, and then processed for immunohistochemistry as above. To determine if neurogenesis occurred on any postnatal days (P1, P3, or P7), P10 sections were co-immunostained for BrdU and NeuN as above except using pretreatment with 2 N HCl at 37°C for 30 minutes in lieu of antigen retrieval. Sections were imaged on a confocal and the pattern of BrdU and NeuN immunolabeling was analyzed.

## Supporting Information

Figure S1 **4A4+/Pax6+ mitotic cells with ventricular and tangential oriented processes are present in macaque cortex.** (**a**) Coronal section from E80 macaque immunostained for 4A4 (green), Pax6 (red) and counterstained with DAPI (blue). The cells indicated by boxes (1 and 2) show Pax6+ mitotic cells with a single process oriented toward the ventricular surface. These cells appear to be unipolar. (**b**) Coronal section from E80 macaque immunostained for 4A4 (green), Pax6 (red) and counterstained with DAPI (blue). The cell highlighted with the box shows a 4A4+ mitotic cell that expresses Pax6 and possesses a short tangential process. VZ, ventricular zone; iSVZ, inner subventricular zone; oSVZ, outer subventricular zone.
(TIF)
