# Supplementary figures and images for: Comparative Analysis of the Subventricular Zone in Rat, Ferret and Macaque: Evidence for an Outer Subventricular Zone in Rodents
Source: PLoS One. 2012 Jan 17;7(1):e30178. doi: 10.1371/journal.pone.0030178 (PMC3260244; doi:10.1371/journal.pone.0030178)

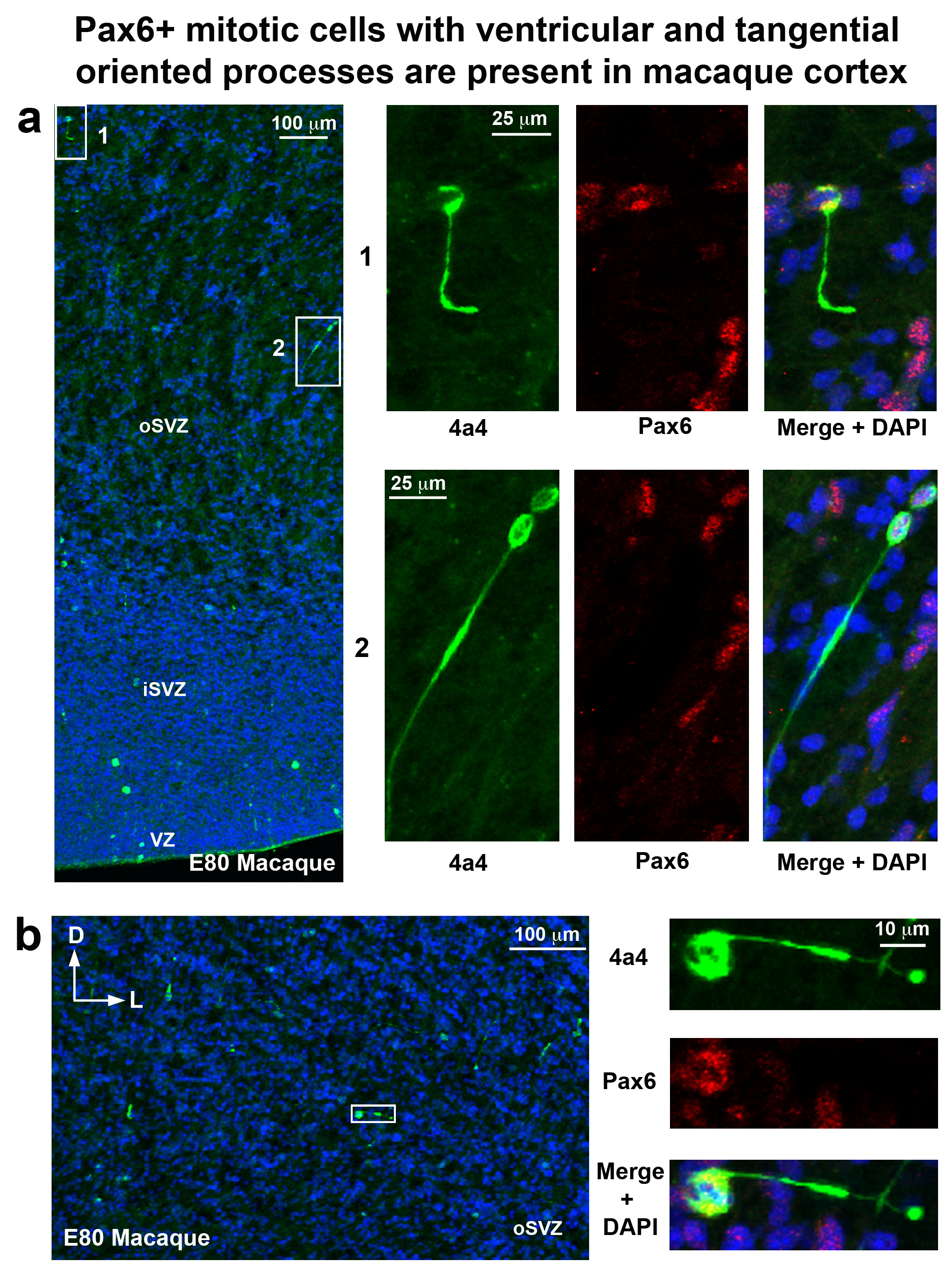

Supplement: Figure S1 — 4A4+/Pax6+ mitotic cells with ventricular and tangential oriented processes are present in macaque cortex. (a) Coronal section from E80 macaque immunostained for 4A4 (green), Pax6 (red) and counterstained with DAPI (blue). The cells indicated by boxes (1 and 2) show Pax6+ mitotic cells with a single process oriented toward the ventricular surface. These cells appear to be unipolar. (b) Coronal section from E80 macaque immunostained for 4A4 (green), Pax6 (red) and counterstained with DAPI (blue). The cell highlighted with the box shows a 4A4+ mitotic cell that expresses Pax6 and possesses a short tangential process. VZ, ventricular zone; iSVZ, inner subventricular zone; oSVZ, outer subventricular zone. (TIF) [file pone.0030178.s001.tif]
